# 3D printing of inherently nanoporous polymers via polymerization-induced phase separation

**DOI:** 10.1038/s41467-020-20498-1

**Published:** 2021-01-11

**Authors:** Zheqin Dong, Haijun Cui, Haodong Zhang, Fei Wang, Xiang Zhan, Frederik Mayer, Britta Nestler, Martin Wegener, Pavel A. Levkin

**Affiliations:** 1 Institute of Biological and Chemical Systems-Functional Molecular Systems (IBCS-FMS) Karlsruhe Institute of Technology, 76344 Eggenstein-Leopoldshafen, Germany; 2 Institute of Applied Materials - Computational Materials Scsience (IAM-CMS), Karlsruhe Institute of Technology, 76131 Karlsruhe, Germany; 3 Institute for Micro Process Engineering (IMVT), Karlsruhe Institute of Technology, 76344 Eggenstein-Leopoldshafen, Germany; 4 Institute of Nanotechnology and Institute of Applied Physics, Karlsruhe Institute of Technology, 76128 Karlsruhe, Germany

**Keywords:** Biomaterials - cells, Polymers, Design, synthesis and processing

## Abstract

3D printing offers enormous flexibility in fabrication of polymer objects with complex geometries. However, it is not suitable for fabricating large polymer structures with geometrical features at the sub-micrometer scale. Porous structure at the sub-micrometer scale can render macroscopic objects with unique properties, including similarities with biological interfaces, permeability and extremely large surface area, imperative inter alia for adsorption, separation, sensing or biomedical applications. Here, we introduce a method combining advantages of 3D printing via digital light processing and polymerization-induced phase separation, which enables formation of 3D polymer structures of digitally defined macroscopic geometry with controllable inherent porosity at the sub-micrometer scale. We demonstrate the possibility to create 3D polymer structures of highly complex geometries and spatially controlled pore sizes from 10 nm to 1000 µm. Produced hierarchical polymers combining nanoporosity with micrometer-sized pores demonstrate improved adsorption performance due to better pore accessibility and favored cell adhesion and growth for 3D cell culture due to surface porosity. This method extends the scope of applications of 3D printing to hierarchical inherently porous 3D objects combining structural features ranging from 10 nm up to cm, making them available for a wide variety of applications.

## Abstract (web summary)

3D printing offers flexibility in fabrication of polymer objects but fabrication of large polymer structures with micrometer-sized geometrical features are challenging. Here, the authors introduce a method combining advantages of 3D printing and polymerization-induced phase separation, which enables formation of 3D polymer structures with controllable inherent porosity.

## Introduction

3D printing offers great flexibility in the fabrication of three-dimensional objects with highly complex geometries, allowing exciting properties and intriguing functionalities previously inaccessible^1^. Polymers are by far the most utilized materials for 3D printing^2^. In most of the cases, the 3D printed polymer material has been non-porous or with porosity “printed” in the form of macroscopic geometrical features. Materials with sub-micrometer porous structures are, however, important for applications in adsorption, separation, or biomedical engineering, due to their unique properties such as high surface-to-volume ratio, selective permeability at the molecular level, and similarities with biological interfaces^3,4^.

Unfortunately, contemporary 3D printing methods are not suitable for fabricating large polymer structures with geometrical features at the sub-micrometer scale. For all 3D printing technologies, there is a trade-off between the printing voxel, building volume, and printing time^5^. Therefore, direct printing of a macroscopic polymer object with nanoporosity (1–100 nm) is currently out of reach. To address this challenge, recent studies circumvented the time-consuming printing of nanoscale pores by using inks containing particle-stabilized nanoemulsions^6^ or polymers with intrinsic microporosity^7^. The former relies on the removal of sacrificial templates while the latter employs free volume originated from rigid and contorted molecular structures to achieve nanoporosity in 3D printed polymers. However, these approaches are not compatible with photopolymerization based 3D printing, thus suffering from the limited geometrical complexity and feature resolution of extrusion-based printings^8^.

Self-assembly offers a distinct pathway to create nanoporous materials via autonomous organization of components into structured patterns^9^. One such approach is polymerization-induced phase separation, which gives rises to a polymer-rich phase to form the porous matrix and a polymer-poor phase to afford porosity^10^. Traditionally, this principle is realized by casting^11^ or molding^12^ of a phase-separating solution to process the materials into a manageable macroscopic form. The produced nanoporous polymers possessing high surface area and permeability are very useful as filtration membranes, chromatography monoliths, or functional coatings^11–13^. However, such materials are generally confined to simple macroscopic geometries and homogenous porous properties, limiting their functionality and applications.

In this study, we introduce a method combining advantages of 3D printing via digital light processing (DLP) and polymerization-induced phase separation, which enables the formation of 3D polymer objects with vast design flexibility at the macroscopic scale, and at the same time, with controllable inherent porosity at the sub-micrometer scale. Recently, polymerization-induced phase separation has been used in 3D printing of multi-component glasses^8^. In that system, phase separation leads to a 3D bi-continuous structure of the organic polymer and preceramic polymer, which is pyrolyzed to form a porous ceramic that can be further sintered into transparent glasses. Polymerization-induced phase separation was also utilized in combination with the two-photon direct laser writing (DLW) method to create porous nanometer to micrometer 3D structures^14^. Although well-known for its superb resolution in 3D microfabrication, two-photon DLW is impractically slow for fabricating large objects due to the inherent competition between printing speed and printing resolution^15^.

Herein, we exploit polymerization-induced phase separation to 3D print complex-shaped macroscopic polymer structures from 100 μm up to several centimeters with controlled inherent nanoporosity. Compared to their inorganic counterparts, polymers are much richer in chemistry and functionality, making these inherently nanoporous 3D polymers valuable for broad applications from adsorption, catalysis, separation to energy storage, tissue engineering, and biomedical applications^16^. As a proof of concept, we show that produced hierarchical polymers combining nanoporosity with micrometer-sized pores demonstrate significantly improved adsorption performance for dye removal due to superior pore accessibility and favored cell adhesion and growth on the nanoporous polymer surface.

## Results

### Working principle

**Fig. 1:**
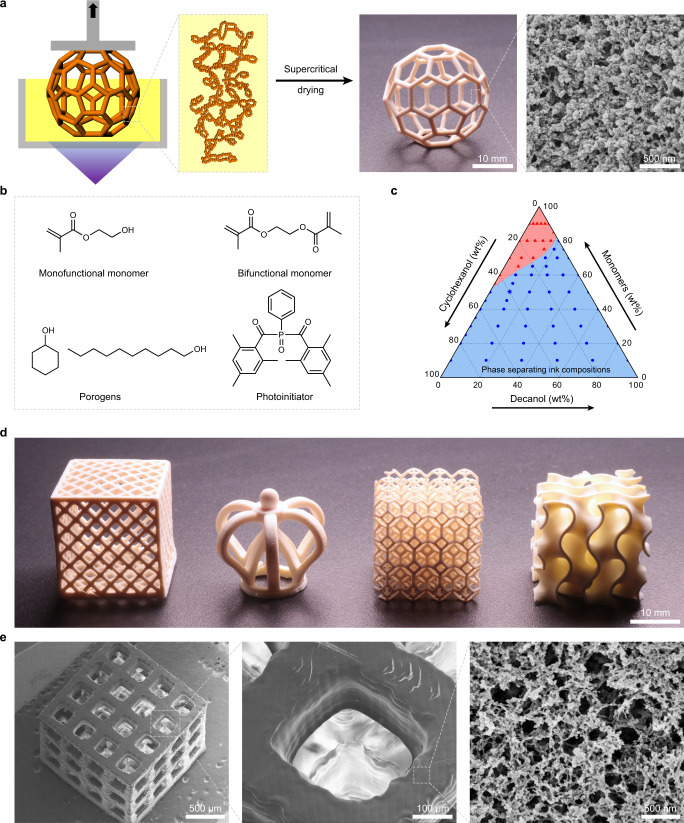
3D Printing of polymer objects with complex macroscopic 3D geometry and defined nanoporous structure. **a** Working principle of the printing process, a photograph of a 3D printed object and an inset cross-sectional SEM micrograph showing the inherent sub-micrometer porous structure. The SEM image shown is a representative of five replicates (*n* = 5) over three independent experiments (*N* = 3). **b** Chemical structures of the ink components. HEMA and EDMA as the monofunctional and bifunctional monomer, cyclohexanol and 1-decanol as the porogen, and Irgacure 819 as the photoinitiator. **c** Ternary diagram showing the initial ink compositions that undergo phase separation upon photopolymerization (the ratio of EDMA to HEMA in the monomer is fixed at 2:3 (w/w)). Blue (symbols and background): the ink phase separated upon photopolymerization and became non-transparent; Red (symbols and background): the ink did not phase separate upon photopolymerization and remained transparent. The blue star indicates the composition of an exemplary ink used for 3D printing of the inherently nanoporous 3D structures illustrated in this figure. **d** Photographs of diverse 3D printed structures: a hollow mesh box, a crown, a lattice cube and a gyroid. **e** SEM micrographs of a 3D printed lattice with 250 μm square pores; an inset showing the inherent sub-micrometer porous surface.

DLP is a 3D printing method that employs projected light patterns to achieve localized polymerization in a vat of ink. Unlike conventional stereolithography based on point-source illumination, DLP enables an entire layer to be cured at once, thus allowing significantly faster printing speed and larger building volume^15^. Typically, the inks used in DLP mainly consist of monomers and/or crosslinkers which polymerize to form the body of a dense 3D object, and a photoinitiator for initiating the polymerization^2^. In order to create inherently nanoporous 3D printed objects, we introduced porogens miscible with the monomers but immiscible with the final polymers into the ink (Fig. 1a).

To illustrate the working principle, we selected an ink consisting of hydroxyethyl methacrylate (HEMA) and ethylene glycol dimethylacrylate (EDMA) as the monofunctional and bifunctional monomer, respectively, a mix of cyclohexanol and 1-decanol as the porogen, and Irgacure 819 as the photoinitiator (Fig. 1b). HEMA was selected as the monomer because of its relatively fast polymerization rate^17^ and well-known biocompatibility useful for biomedical applications^18,19^. Cyclohexanol and 1-decanol were selected as the porogens because both solvents are fully miscible with the monomers, and have both low evaporation rate and viscosity essential for DLP 3D printing^20^. Irgacure 819 was selected as the photoinitiator based on its absorption spectrum matching the emission spectrum of the DLP printer used (Supplementary Fig. 1). For successful 3D printing of inherently nanoporous structures, the ink should (1) undergo phase separation upon photopolymerization, (2) lead to a mechanically stable 3D structure. Therefore, we first measured the light transmittance of a 75 μm thick film prepared by photopolymerization of inks with a range of monomer and porogen compositions (Supplementary Note 3). The decrease in light transmittance was then used as an indication of phase separation to construct the ternary diagram (Fig. 1c), which demonstrates compositions of ink mixtures that can phase separate upon photopolymerization. We also compared the mechanical properties of 3D structures printed using inks with different porogen contents. Clearly, increasing porogen content in the ink leads to 3D structures with a lower density, and therefore, a decrease in their mechanical strength (Supplementary Fig. 3).

Based on these considerations, we selected an exemplary ink consisting of 30 wt% HEMA, 20 wt% EDMA (0.44 molar ratio to HEMA), 40 wt% cyclohexanol, 10 wt% 1-decanol, and 4 wt% Irgacure 819 with respect to monomers (Fig. 1c). The ink was introduced to the DLP printer, where the 3D object was built in a layer-by-layer manner: by moving the build platform in the vertical direction, each layer was successively irradiated by patterned UV light for a predefined time to trigger localized photopolymerization and phase separation of a polymer-rich phase from the polymer-poor phase. Having completed the printing process, the printed 3D object was immersed in acetone for 24 h to remove unreacted monomers and porogens, followed by CO_2_ supercritical drying (see Methods for details).

The obtained 3D objects feature a porous structure at sub-micrometer scale, and a white appearance due to light scattering (Fig. 1a). Using the novel ink, we fabricated four objects including a hexagonal mesh box (hollow), a crown (overhang), an intricate cube (lattice), and a gyroid (curvature) (Fig. 1d). This demonstrates the capability of our method to produce inherently nanoporous objects with highly complex geometries that are difficult to achieve by extrusion or molding.

The printing resolution was investigated by printing an array of pillars with different diameters and 1 mm height. Pillars with diameters above 100 μm can be successfully printed using a simple desktop DLP printer, indicating a minimum printing feature size of ~100 μm (Supplementary Note 4). The achieved printing resolution is comparable to those of most commercially available DLP 3D printers^21^. With this resolution, we were able to print a cube lattice with pores of 250 μm (Fig. 1e). The printing resolution might be further improved by using custom-designed printer or direct laser writing methods with higher resolutions^14,22^.

**Fig. 2:**
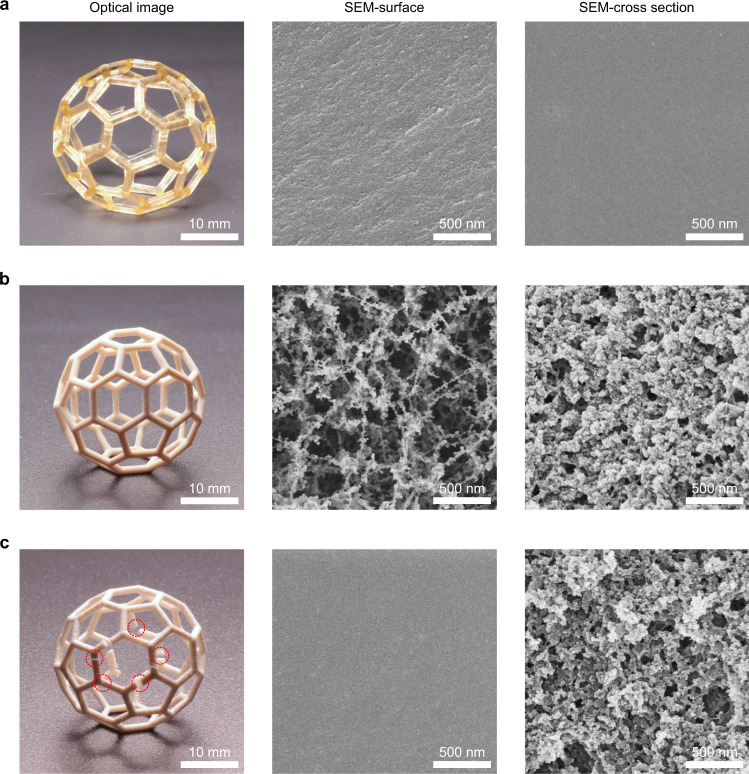
Supercritical drying for preserving the structural integrity and complete porosity of the inherently nanoporous 3D objects. Photographs and SEM micrographs of (**a**) a non-porous 3D object printed using an ink without porogens (60 wt% HEMA, 40 wt% EDMA, 4 wt% Irgacure 819 with respect to monomers) followed by supercritical drying, (**b**) an inherently nanoporous 3D object printed using an ink with porogens (30 wt% HEMA, 20 wt% EDMA, 40 wt% cyclohexanol, 10 wt% 1-decanol, 4 wt% Irgacure 819 with respect to monomers) followed by supercritical drying, and (**c**) a 3D object printed using the ink with porogens followed by air drying, with cracks visible on the photograph. The surface and cross-sectional SEM images shown are representative of three replicates (*n* = 3) over three independent experiments (*N* = 3).

Polymerization-induced phase separation plays a key role in the generation of the inherent nanoporous structures. As shown in Fig. 2a, 3D objects printed using a conventional ink containing only monomers but without porogens are completely non-porous and transparent. This is in sharp contrast with the opaque, inherently nanoporous 3D objects printed using the phase-separating ink (Fig. 2b). It is worth noting that the supercritical drying step is crucial to ensure a complete nanoporosity through the 3D printed objects, as drying them in air results in cracked objects with a non-porous surface shell (Fig. 2c). As revealed by the SEM micrographs, the supercritically dried 3D objects exhibit different microstructure inside and at the surface: the surface shows a more open microstructure with smaller globules (Fig. 2b). This difference is attributed to the open environment of the DLP printer used, as it uses an oxygen-permeable window to create a “dead zone” for preventing adhesion between the window and the emerging printed part^23^. This results in a higher oxygen concentration at the surface of the curing layer, leading to lower conversion of monomers, a well-known phenomenon in free-radical photopolymerization^24^. The low monomer conversion further leads to a smaller globule size and a more porous microstructure at the surface of 3D printed objects, and may lead to a poorer mechanical strength^25,26^. When dried in air, the 3D printed objects experience a strong capillary force, leading to the collapse of the surface pores and formation of cracks^27^. In contrast, supercritical drying can avoid liquid-vapor transition during solvent elimination^28^, allowing the 3D printed objects to maintain their structural integrity and surface porosity.

A salient feature of nanoporous materials is their low density. Indeed, the nanoporous 3D polymers show a density of 0.82 g cm^−3^, which is 69% of their non-porous counterparts (1.19 g cm^−3^). The density of the 3D polymers can be further reduced to 0.35 g cm^−3^ (relative density 29%) by 3D printing of a hierarchically porous structure. Although the compressive strength of the 3D structures inevitably decreases from 201.6 MPa (non-porous) to 40.3 MPa (nanoporous) to 6.1 MPa (hierarchically porous) due to decreased density, the 3D hierarchically porous structures still have a sound mechanical stability and can be handled without special care (Supplementary Fig. 5). As the mechanical strength of macroscopic materials is highly dependent on their microarchitecture^29^, by exploiting the design flexibility of DLP 3D printing, this method can potentially be used for the 3D printing of extremely lightweight materials.

### Control of the inherent nanoporous structures

Controlling the pore size of nanoporous polymers is important, for example, for applications in liquid filtration^30^, or cell culture^31^. One advantage of our method lies in its flexibility to control the inherent nanoporous structures in 3D printed objects by adjusting processing parameters including ink compositions or irradiation intensity^32^. We demonstrate such possibility by tuning the porogen composition in the inks while keeping the monomer/porogen ratio constant. Five ink mixtures with increasing ratio of 1-decanol/cyclohexanol from Mix-1, containing pure cyclohexanol as a porogen, to Mix-5, containing pure 1-decanol as a porogen, were prepared according to Table 1. The viscosity of these inks experiences a slight decrease from 8.6 mPa s for Mix-1 to 6.1 mPa·s for Mix-5 (Supplementary Fig. 6a), remaining at a low level suitable for DLP 3D printing. On the other hand, the curing rate of the inks gradually decreases with increasing 1-decanol concentration, which can be attributed to the different solvation effects of cyclohexanol and 1-decanol^33^.

**Table 1:** Physical properties of 3D printed polymers possessing sub-micrometer scale porous structures.

Mixture number	HEMA (wt%)	EDMA (wt%)	1-Decanol (wt%)	Cyclo hexanol (wt%)	Average pore size^a^ (nm)	Monomer Conversion^b^ (%)	Linear shrinkage^c^ (%)	Surface area^d^ (m^2^ g^−1^)	Density^e^ (g cm^−3^)	Young’s modulus^f^ (MPa)	Compressive strength^g^ (MPa)
1	30	20	0	50	24 ± 5	99.1 ± 0.7	16.8 ± 1.0	53.5 ± 1.7	0.97 ± 0.03	428. 0 ± 8.0	56.2 ± 4.2
2	30	20	10	40	49 ± 5	96.6 ± 0.8	13.0 ± 0.6	44.1 ± 1.0	0.82 ± 0.02	268.0 ± 3.1	40.3 ± 2.0
3	30	20	25	25	91 ± 15	87.2 ± 1.1	8.4 ± 0.3	34.1 ± 1.6	0.60 ± 0.02	70.0 ± 1.6	22.5 ± 0.5
4	30	20	40	10	405 ± 88	66.2 ± 0.8	6.2 ± 0.2	7.9 ± 0.2	0.42 ± 0.01	20.2 ± 0.6	5.3 ± 0.1
5	30	20	50	0	548 ± 119	61.4 ± 1.4	7.4 ± 0.7	6.1 ± 0.6	0.38 ± 0.01	10.5 ± 0.3	2.6 ± 0.1

The data represents the mean ± s.d. of three independent experiments (*N* = 3).

^a^Average pore size measured from the SEM cross-section images using ImageJ.

^b^Monomer conversion calculated from the weight of 3D printed cubes (5 × 5 × 5 mm^3^) as compared to the weight of monomer (5 × 5 × 5 mm^3^) in the ink solution.

^c^Linear shrinkage calculated from the length of 3D printed cubes (5 × 5 × 5 mm^3^) before and after drying using optical microscopy.

^d^Surface area calculated from N_2_ adsorption isotherm using the BET equation.

^e^Density measured from the weight and volume of 3D printed cubes (5 × 5 × 5 mm^3^).

^f^Young’s modulus calculated from the stress–strain curve of 3D printed cubes (5 × 5 × 5 mm^3^).

^g^Compressive strength calculated from the stress–strain curve of 3D printed cubes (5 × 5 × 5 mm^3^).

**Fig. 3:**
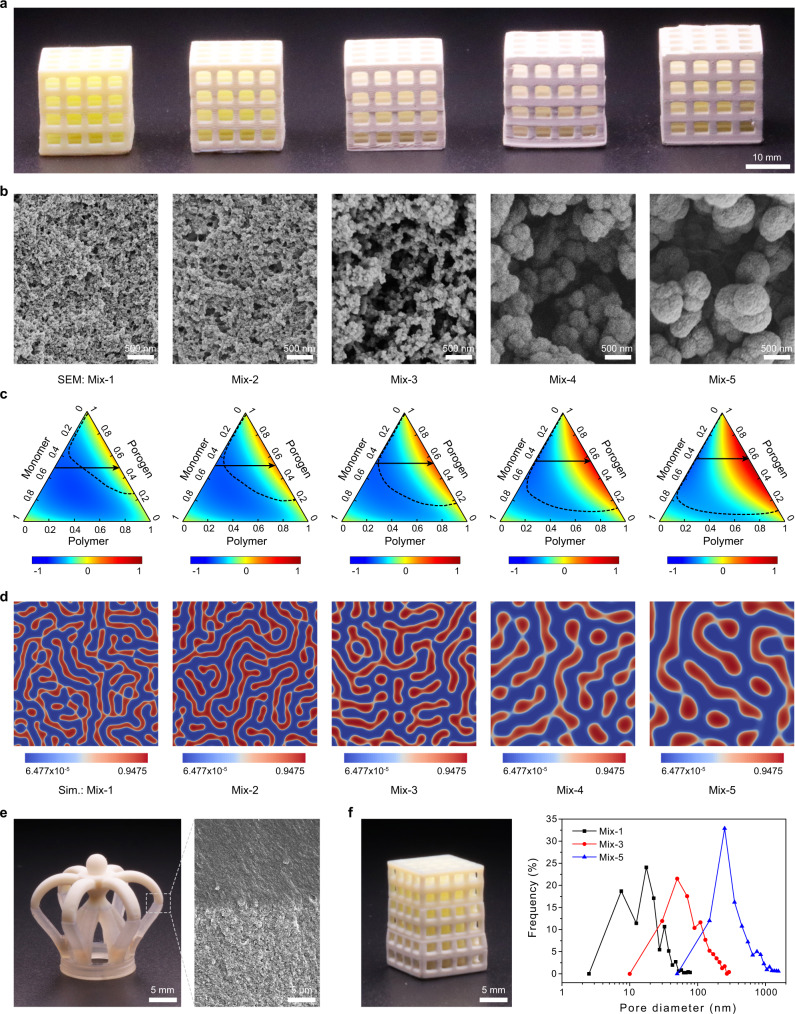
Control of the inherent nanoporous structures in 3D printed objects. **a** Photographs of 3D printed lattices using five inks with different porogen compositions (Mix-1 to Mix-5 from left to right). **b** Cross-sectional SEM micrographs of 3D polymer cubes (5 × 5 × 5 mm^3^) printed using the five inks. **c** Phase diagrams of the five inks based on the Flory–Huggins model (Mix-1 to Mix-5 from left to right). The magnitude of the free energy of the mixture for the system is demonstrated with the gradient color and normalized by its maximum value, which acts the main driving force for the phase separation. The dashed line represents the spinodal line, and the solid arrow represents the polymerization process. The intersection indicates the point of the phase separation. **d** Simulated microstructures with the five inks using the phase-field method coupling with the Flory-Huggins model (red: polymer-rich phase; blue: solvent-rich phase). **e** A photograph of a 3D object with bi-disperse porosity, printed by using an ink with 50 wt% porogens (Mix-2) for the upper part and an ink without porogens (Mix-6) for the lower part; an inset cross-sectional SEM micrograph showing the sharp interface. **f** A photograph of a 3D object with tri-disperse pore size distributions, printed by using ink Mix-1 for the upper part, ink Mix-3 for the middle part and ink Mix-5 for the lower part. The SEM images shown in (**b**, **e**) are representative of three replicates (*n* = 3) over three independent experiments (*N* = 3).

Based on the five inks, 3D lattice cubes with distinct nanoporous structures were successfully printed (Fig. 3a, b). As illustrated by the cross-sectional SEM micrographs, with 1-decanol concentration increasing from 0 wt% to 50 wt% in the ink, the average pore size of the inherently nanoporous 3D polymers gradually increases from 24 nm to 548 nm. Such a difference is mainly attributed to the distinct solvation effect of cyclohexanol and 1-decanol. To show the influence of porogen composition on the phase separation microstructures, we used the mean-field Flory–Huggins theory to compute the conversion phase diagram of the system and simulated the microstructure evolution in polymerization-induced phase separation by adopting a Cahn–Hilliard type phase-field model (Supplementary Note 7). As cyclohexanol is a better solvent than 1-decanol for the polymers, we increased the Flory parameter χ with a rise of 1-decanol concentration in the inks, which corresponds to the increase in the intermolecular repulsive forces between the polymers species and porogen molecules. The phase diagrams show that the spinodal region gradually expands with increasing 1-decanol concentration, which leads to phase separation at an earlier stage of polymerization (Fig. 3c). Also, the phase separation takes place more rapidly with increasing the concentration of 1-decanol, leading to the formation of thicker polymer networks (Fig. 3d), which agrees well with the experimental results (Fig. 3b).

The evolution of the nanoporous structures leads to a dramatic change of the physical properties of the 3D printed objects. The increase of pore size for objects printed from Mix-1 to Mix-5 leads to a decrease of their BET surface area from 53.5 m^2^ g^−1^ to 6.1 m^2^ g^−1^ (Table 1), and a sharp decline of their transparency (Supplementary Fig. 7a). The linear shrinkage ratio of the 3D printed objects decreases from 16.8% for objects printed using Mix-1 to 7.4% for objects printed using Mix-5, due to the weaker capillary force experienced in the supercritical drying process. On the other hand, the monomer conversion of the 3D printed objects drops from 99.1% to 61.4% from Mix-1 to Mix-5, which might be attributed to a reduced curing rate with increasing 1-decanol concentration in the ink (Supplementary Fig. 6b). Consequently, the density of the 3D printed objects decreases from 0.97 g cm^−3^ to 0.38 g cm^−3^, leading to a drastic decrease of their mechanical strength. The Young’s modulus and compressive strength of the 3D printed objects decrease from 428.0 MPa and 56.2 MPa for objects printed using Mix-1 to 10.5 MPa and 2.6 MPa, respectively, for objects printed using Mix-5 (Table 1 and Supplementary Fig. 7b).

As DLP is a layer-by-layer 3D printing technique, we were able to create 3D objects with spatially controlled nanoporous structures by switching the inks during printing. In this way, a 3D object with bi-disperse porosity was successfully fabricated by switching between an ink with 50 wt% porogens (Mix-2) and an ink without porogens (Mix-6, see Supplementary Note 1), with a sharp interface clearly seen between the porous and non-porous parts (Fig. 3e). A 3D lattice with tri-disperse pore size distributions was also produced by switching between ink Mix-1, Mix-3, and Mix-5, with pore size tunable from tens to hundreds of nanometers (Fig. 3f and Supplementary Fig. 8). To check whether switching the inks during 3D printing would generate interfacial defects that may lead to mechanical failure, we tested the mechanical strength of a heterogeneous 3D structure printed using ink Mix-1 and Mix-2. The heterogeneous 3D printed cube demonstrates a compressive strength value between those of the cubes printed using ink Mix-1 and Mix-2 only (Supplementary Fig. 9), suggesting that switching inks does not lead interfacial defects. The flexibility to vertically control the inherent nanoporous structures of 3D objects can be further exploited to create nanoporous polymers with gradient pore size or porosity, which are useful for various biological applications^34–36^.

### Structured adsorbents for pollutant removal

Nanoporous polymers have great potentials in the field of adsorption because of their high surface area and chemical diversity. While extensive studies have centered on improving their adsorption capacity and selectivity, most of the materials are produced and evaluated in the form of powders^37–39^. To realize industrial-scale implementation, it is crucial to shape such adsorbents into a macroscopic form that is manageable while maintaining efficient mass transfer^40^. In this context, our method provides a unique opportunity to design structured adsorbents combining nanoporosity for high surface area with micrometer-sized pores for promoting mass transfer. As a proof of concept, we demonstrate here 3D printing of monolith adsorbents with hierarchical porosity for dye removal from water.

**Fig. 4:**
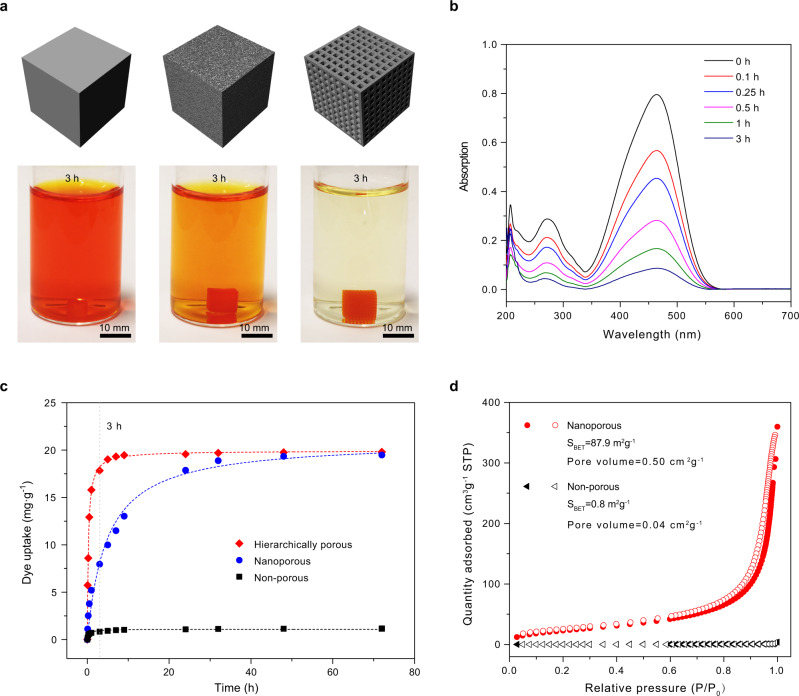
Adsorption of aqueous methyl orange by 3D printed adsorbents. **a** Photographs of 20 mL methyl orange solutions (100 ppm) after incubating 20 mg of the non-porous, nanoporous, and hierarchically porous adsorbents in the solution for 3 h. **b** Time-dependent UV–Vis spectra of the methyl orange solution in the presence of the hierarchically porous adsorbent (analytes were diluted by a factor of 10). **c** Time-dependent methyl orange uptake by the non-porous, nanoporous, and hierarchically porous adsorbents in 72 h, with pseudo-second order kinetic modeling (dash lines). **d** N_2_ adsorption (solid) and desorption (hollow) isotherms of the inherently nanoporous polymer and the non-porous polymer. Representative of three independent experiments (*N* = 3).

For 3D printing of the monolith adsorbents, dimethyl aminoethyl methacrylate (DMAEA) was selected as the monofunctional monomer, for its tertiary amino group could bind to various anionic dyes through electrostatic interactions. DMAEA cures slower than HEMA (Supplementary Fig. 10), and therefore requires a longer cure time in the DLP printing process (Supplementary Note 1). Three polymer cubes (adsorbents) based on the same amount (≈20 mg) of the same amino-functionalized polymer but with different structures were printed: a non-porous cube (5.5 × 5.5 × 5.5 mm^3^), a nanoporous cube (6.8 × 6.8 × 6.8 mm^3^) and a hierarchically micro-nano porous cube (9.4 × 9.4 × 9.4 mm^3^ lattice with 300 μm thick walls) (Fig. 4a). The nanoporous and hierarchically porous cubes were printed using an ink with porogens (Mix-7), while the non-porous cube was printed by using an ink without porogens (Mix-8, see Supplementary Note 1). The kinetics of uptake of methyl orange (MO), a model dye, into those adsorbents was assessed by adding adsorbents (1 mg ml^−1^) into 20 ml of MO aqueous solution (100 ppm). As shown in Fig. 4c, the nanoporous adsorbent demonstrates a much higher equilibrium uptake (21.00 mg g^−1^) than the non-porous adsorbent (1.09 mg g^−1^). This is attributed to the open porosity of the nanoporous adsorbent (Supplementary Fig. 11), leading to a large surface area of 87.9 m^2^ g^−1^, 100-fold higher than that of the non-porous adsorbent (0.8 m^2^ g^−1^) (Fig. 4d). The hierarchically porous adsorbent shows an equilibrium uptake comparable to the nanoporous adsorbent, but with a 16-fold increase in the apparent pseudo-second-order rate constant (Supplementary Table 2). The rapid kinetic uptake of the hierarchically porous adsorbent is attributed to its much smaller characteristic length (150 μm) when compared to the nanoporous adsorbent (3.4 mm), which significantly reduces diffusion time and improves dye transfer efficiency. The hierarchically porous adsorbent could remove 90% of the dye in 3 h, while the nanoporous and non-porous adsorbents require more than 24 h (Fig. 4a–c), demonstrating the advantage of hierarchical structure at the micro-nano scale created by 3D printing.

3D printing offers an exciting platform to formulate nanoporous materials into monolith adsorbents with highly tunable structure and geometries^41^. Earlier efforts in this direction have enabled 3D printing of Metal-organic frameworks (MOFs)^42,43^, a porous inorganic-organic hybrid material with extremely large surface area (thousands of m^2^ g^−1^). Such 3D printed MOF monoliths demonstrate adsorption capacity comparable to MOF powders but with faster adsorption kinetics^42^. Here, our method complements these developments by enabling 3D printing of polymers with nanoporosity built into it by polymerization-induced phase separation. Although the surface area of the 3D printed nanoporous polymers is lower than that of their MOF counterparts, the advantage of our method lies in the increased tunability of the monomers and the control over porosity via phase separation.

### Inherently nanoporous 3D scaffolds for cell culture

3D printing has recently attracted great interest in the field of tissue engineering, for its ability to create biological scaffolds with intricate architectures for guiding cell growth in three dimensions, resembling the complex architecture of organs^44^. To date, most 3D printed scaffolds used for cell seeding feature a non-porous body structure and a smooth surface, which may lead to insufficient cell attachment and requires further functionalization with adhesive moieties to enhance cell adhesion^45–47^. Scaffold materials with nanoporous topography have shown significantly improved cell attachment compared to their non-porous counterparts^48–50^. Here we demonstrate that 3D printed polymer scaffolds could benefit from the introduction of inherent nanoporosity to promote cell adhesion and, as a consequence, to increase the cell density on the surface of printed structures.

**Fig. 5:**
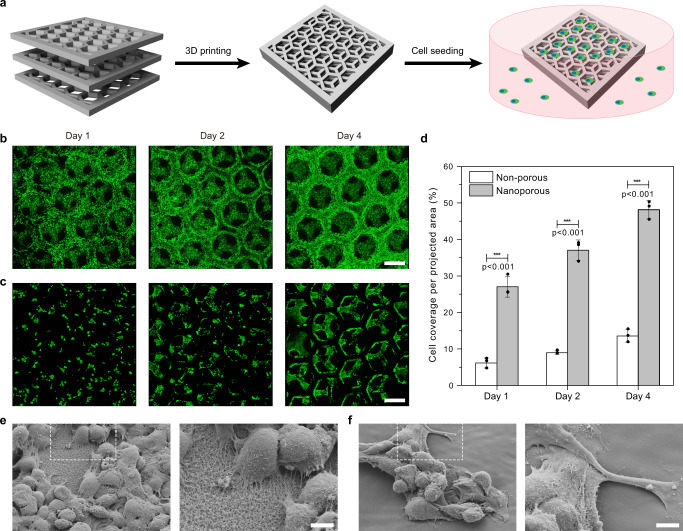
Hierarchical inherently nanoporous scaffolds with improved biocompatibility for 3D cell culture. **a** Schematic representation of the scaffolds´ geometry. **b**, **c** 3D confocal microscopy images of Hep G2 cells cultured on the inherently nanoporous scaffold (**b**) and the non-porous scaffold (**c**) after 1, 2, and 4 days of culture (day 1, day 2 and day 4 from left to right). Scale bars: 500 μm. The 3D confocal images were integrated from 30 z-stack images with a single stack thickness of 10 µm. **d** Coverage of live cells (Calcein-positive) per projected area per projected area calculated from the 3D confocal images within a volume of 3 × 3 × 0.3 mm^3^. Error bars are standard deviations of three independent experiments (*N* = 3). The statistical significance was assessed using unpaired two-tailed Student’s *t* test. **e**, **f** Overview and zoom-in SEM micrographs of Hep G2 cells cultured on the inherently nanoporous scaffold (**e**) and the non-porous scaffold (**f**) after 1 day. Scale bars: 5 μm. The SEM images shown are representative of three replicates (*n* = 3) over three independent experiment (*N* = 3).

For 3D printing of scaffolds for cell culture, we used HEMA as the monofunctional monomer because of its biocompatibility^18^. 3D hexagonal scaffolds (Fig. 5a) with and without inherent nanoporosity were printed using ink Mix-2 and Mix-6 (Supplementary Note 1), respectively. To evaluate the capability of the scaffolds for supporting cell adhesion and proliferation, Hep G2 cells (1 × 10^6^ cells per mL) were seeded on the inherently nanoporous and non-porous 3D scaffolds, and were analyzed by fluorescence confocal microscopy 1, 2, and 4 days post-seeding. The surface coverage of the scaffolds during the culture period was quantified by analyzing the integrated 3D images. Results show that the area covered by live cells (Calcein-positive) on the inherently nanoporous 3D scaffold (27.0%) was 4 times higher than that of the non-porous 3D scaffold (6.1%) after 1 day (Fig. 5b–d), indicating favored initial cell adhesion. The cell coverage on the inherently nanoporous 3D scaffold gradually increased with the extended incubation period and remained 3-fold higher than that of the non-porous 3D scaffold (Fig. 5b–d). In addition, we observed high viability (~95%) of the cells on the scaffolds during the culture period, with only a few dead cells (PI-positive) (Supplementary Fig. 13).

The reason for the enhanced cell adhesion on the nanoporous 3D scaffolds might be multifold. The nanoporous scaffolds possess a larger specific surface area available for cells to adhere. The nanoporous topography might alter cell anchorage, allowing more filopodia to anchor more tightly^49–52^. As evidenced by immunostaining and SEM microscopy, the Hep G2 cells have more protrusions on the inherently nanoporous 3D scaffold (Supplementary Fig. 14 and Fig. 5e, f). On the other hand, culturing of cells in a serum-free medium resulted in a similar two-fold difference in cell adhesion between nanoporous vs. non-porous scaffolds (Supplementary Fig. 15), indicating that non-specific protein adsorption might not be the main reason for the dissimilar cell adhesion. To prove that the surface nanoporosity is indeed beneficial for cell attachment, we seeded Hep G2 cells (1 × 10^6^ cells per mL) on a nanoporous and a non-porous flat polymer layer without any 3D geometry. After 1 day culturing, the cell coverage on the nanoporous plate was 30 times higher than that of the non-porous plate (Supplementary Fig. 16), confirming the efficacy of surface nanoporosity in improving cell adhesion. Hence, the inherently nanoporous 3D scaffolds hold great promise for 3D cell culture applications in tissue engineering.

## Discussion

3D printing has opened a new era in the development of functional materials by offering almost unlimited freedom to manipulate geometric shapes. Realization of its ultimate potential requires not only the advances of printing techniques, but also the development of new materials leading to objects with novel physicochemical properties. 3D printing can greatly benefit from the ability to print 3D objects possessing sub-micrometer porous structures, which provides unique properties including similarities with biological interfaces, permeability, and extremely large surface area, important for applications in gas storage, water treatment, liquid chromatography, or biomedical engineering. However, direct 3D printing of a macroscopic polymer object with nanoporosity is currently not possible due to the inherent competition between printing speed and printing resolution.

Here, we propose a solution to this challenge by combining top-down 3D printing of macroscopic objects with bottom-up self-organization of the polymer into nanoscale porous structures. This marriage allows independently controlled macrostructure at the sub-millimeter scale defined by DLP 3D printing and nanostructure at the sub-micrometer scale regulated by self-assembly (phase separation). We demonstrate the possibility to create 3D polymer structures of highly complex geometries and spatially controlled pore sizes from 10 nm to 1000 µm. Such hierarchically structured polymers, combining the advantages of extremely high surface area, perfect pore accessibility and complex geometry, demonstrate improved adsorption performance and favored cell adhesion for 3D cell culture. Importantly, the monomers that can be used are diverse, affording the inherently nanoporous 3D polymers with versatile chemical functionality for broad applications from adsorption, filtration, catalysis to drug delivery and tissue engineering.

Although our method offers significant advantages, several limitations remain. This includes the inherent trade-off between the porosity and mechanical strength of the 3D printed objects, and the requirement of supercritical drying to keep structure integrity and surface porosity, as well as the need to remove porogens from the polymerized material. In addition, there is an inherent distribution of the pore sizes and polymer globules, which might be a limitation for specific applications.

## Methods

### Ink preparation and characterization

Irgacure 819 was purchased from Ciba and all other chemicals were all bought from Sigma-Aldrich. The monomers were purified by a short column filled Al_2_O_3_ to remove inhibitors before usage. The inks used for 3D printing were prepared by mixing a certain amount of monomers, porogens, and photointitors. The inks were sonicated for 30 min to obtain a clear and homogenous solution, and then stored in 4 °C fridge before usage. The compositions for different inks used in this study are listed in Supplementary Note 1.

The rheological property of the inks was measured by a rheometer (Thermo Fisher Scientific, HAAKE Rheostress 1). Apparent viscosity was measured as a function of shear rate within a sweep of shear rate (1–150 s^−1^).

To determine the working curve, the ink was illuminated in the DLP 3D printer with a 1 × 1 mm^2^ image for a certain time. The depth of the cured structure was then measured using a droplet shape analyzer (Krüss DSA 25).

### 3D printing

A commercial desktop DLP printer (Miicraft Plus) was used for all the 3D printing experiments. The setup of the 3D printer is shown in Supplementary Fig. 17. The printer is based on a LED projector (405 nm) with an intensity of 1.0 mW cm^−2^ at the vat and a resolution of 450 ppi (∼56 μm). The build area is 43 mm × 27 mm × 180 mm and the layer thickness is adjustable from 5 to 200 μm.

Before printing, the ink (~10 mL) was poured into the resin tank. The 3D printing started by irradiating the ink with a pre-determined cure time for a given layer thickness (Supplementary Note 1). After printing, the 3D printed objects were carefully separated from the build platform and then immersed in acetone for 24 h to remove unreacted monomers and porogens. The volume change of the 3D printed nanoporous polymers in acetone was almost negligible (Supplementary Fig. 18), ensuring the structural fidelity of the 3D printed objects.

For drying of 3D printed objects, supercritical drying was used to avoid the collapse of the nanoporous structures. The printed objects immersed in acetone were first transferred to the chamber of the supercritical apparatus (Leica EM CPD030), followed by exchanging the solvent with CO_2_ at 10 °C five times (each exchange took ~1 min), reaching a pressure of 50 bar. Then, the chamber temperature and pressure were increased to 35 °C and 90 bar in 15 min to maintain the CO_2_ in supercritical condition. Finally, the chamber pressure was gradually dropped to atmospheric pressure for releasing the CO_2_.

### Characterization of 3D printed objects

The pore structure of the 3D printed objects was characterized by a scanning electron microscope (Zeiss LEO 1530) at an operating voltage of 5 kV. Prior to the SEM measurements, the samples were coated with a 7 nm thick platinum layer. Pore sizes were measured from the cross-sectional SEM images by using the ‘Local Thickness’ plugin for Image J (Supplementary Note 9).

The nitrogen sorption of the 3D printed objects was carried out at 77 K with a surface characterization analyzer (Micromeritics 3Flex). Prior to the measurement, the samples were grinded into powders, and then degassed at 343 K under vacuum for 8 h using a SmartVac Prep (Micromeritics). The specific surface area was determined by using the Brunauer–Emmett–Teller (BET) method (Supplementary Note 20).

The UV–Vis transmittance of the 3D printed films (thickness of 75 μm) was measured using a UV−Vis spectrometer (PerkinElmer Lambda 35).

The monomers conversion *α* of the 3D printed cubes (5 × 5 × 5 mm^3^ by design) was calculated from the weight of the printed cubes as compared to the weight of the monomers (5 × 5 × 5 mm^3^) in the ink using the following equation:

1 $$\alpha = \frac{m}{{\rho Vc}},$$

where *m* is the weight of the 3D printed cube, *ρ* is the density of the ink, *V* is the volume of the ink irradiated by UV (125 mm^3^), *c* is the concentration of the monomers in the ink (50 wt%).

The density ρ of the 3D printed cubes (5 × 5 × 5 mm^3^ by design) was calculated using the following equation:

2 $${\uprho} = \frac{m}{{L^3}},$$

where *m* and L are the weight and length of the 3D printed cube, respectively.

The mechanical properties of the 3D printed cubes (5 × 5 × 5 mm^3^ by design) were tested using a universal testing machine (ZwickRoll 2.5 kN). The specimen was compressed in the Z-direction at a rate of 0.5 mm min^−1^ until sample fracture was detected in the stress-strain plot. The Young’s modulus was obtained by calculating the slope of the initial linear region of the stress to strain curves. The strength at the fracture point was used as the compressive strength.

### Pollutant adsorption experiments

Methyl orange (MO), a negatively charged dye, was used as the model pollutant. Three polymer cubes (adsorbents) based on the same amount (≈20 mg) of the same amino-functionalized polymer but with different structures were printed: a non-porous cube (5.5 × 5.5 × 5.5 mm^3^), a nanoporous cube (6.8 × 6.8 × 6.8 mm^3^) and a hierarchically micro-nano porous cube (9.4 × 9.4 × 9.4 mm^3^ lattice with 300 μm thick walls). The nanoporous and hierarchically porous cubes were printed using an ink with porogens (Mix-7), while the non-porous cube was printed by using an ink without porogens (Mix-8, see Supplementary Note 1). The adsorbents were weighed and then put into 100 ml conical shaker flasks (Duran Group) containing 20 mL of 100 ppm MO solution, and the flasks were placed in an incubator shaker (New Brunswick) at 260 rpm and a constant temperature of 20 °C. Samples (0.1 mL) were taken from the flask before adsorbent addition and at certain time intervals. The sampling periods were 0, 0.1, 0.25, 0.5, 1, 3, 5, 7, 9, 24, 32, 48, and 72 h. The MO concentration of the samples was measured by UV-Vis spectroscopy using a UV-Vis spectrometer (PerkinElmer Lambda 35). All samples were diluted by a factor of 10 to allow a linear relationship between concentration and absorbance. The dye uptake of the adsorbents was calculated by the mass balance equation:

3 $$q = \frac{{V \times \left( {c_0 - c_t} \right)}}{m},$$

where *V* is the volume of dye solution, *m* is the mass of the adsorbent, *c*_*0*_ and *c*_*t*_ are the initial concentration and the measured concentration at *t*, respectively.

### Cell culture experiments

In the 3D cell culture experiments, inherently nanoporous 3D scaffolds were printed using an ink with porogens (Mix-2) and non-porous 3D scaffolds were printed using an ink without porogens (Mix-6, see Supplementary Note 1). All the samples were sterilized and degassed before the cell experiments. The suspended Hep G2 cells (1 × 10^6^ cells per mL) were then seeded on the nanoporous and non-porous 3D scaffolds. The cells were cultured in Dulbecco’s modified Eagle’s medium (DMEM, Gibco™, and 41966029) containing 10% fetal bovine serum (FBS, Gibco™, and 10270106) in a standard incubator (5% CO_2_, 37 °C, Thermo Scientific™), and the medium was exchanged every day to avoid additional variability of the results. After the appointed days (1, 2, 4 days), the cells were stained with Calcein-AM (Invitrogen™, 2 µg mL^−1^) and propidium iodide (PI, Invitrogen™, 2 µg mL^−1^) to check the cell viability on the scaffold (the dyes were used according to the manufacturer’s protocols). The samples were then washed with PBS (1X, Gibco™) three times and imaged by a confocal microscope (Zeiss LSM 800). To visualize the dispersion of cells on the 3D structure, the Z-stack technology was used with a scanning range of 300 µm and a single stack thickness of 10 µm. The selected stacks of images were treated by Image J to construct a 3D image. (A 3D plugin could be downloaded from the website, https://imagej.nih.gov/ij/). Cell coverage was calculated by measuring the area of the green fluorescence of the integrated 3D images within a volume of 3 × 3 × 0.3 mm^3^ using the Otsu’s thresholding function in ImageJ, which was divided by the total area of the projected image (3 × 3 mm^2^) to give the “cell coverage per projected area”.

In the 2D cell culture experiments, inherently nanoporous 2D plates were printed using an ink with porogens (Mix-2) and non-porous 2D plates were printed using an ink without porogens (Mix-6). The suspended Hep G2 cells (1 × 10^6^ cells per mL) were seeded on the nanoporous and non-porous 2D plates under the same condition used in 3D cell culture. After 1 day culturing, the cells were stained with Calcein-AM (Invitrogen™, 2 µg mL^−1^) and propidium iodide (PI, Invitrogen™, 2 µg mL^−1^) to check the cell viability on the plates. The samples were then washed with PBS (1X, Gibco™) three times and imaged by a confocal microscope (Zeiss LSM 800). Cell coverage was calculated by measuring the area of the green fluorescence of the 2D images within an area of 1.8 × 1.8 mm^2^ using the Otsu’s thresholding function in ImageJ, and dividing it by the total area.

To check the cell adhesion on the surface, the cells were fixed and observed by a scanning electron microscope (Zeiss LEO 1530) at an operating voltage of 5 kV. After 1 day of cell cultivation on the 3D scaffolds, the samples were washed with PBS (1X, Gibco™) three times and immersed in glutaraldehyde solution (2.5 wt%) for 1 h. The samples were then washed with PBS (1X, Gibco™) three times, and a typical cell dehydration process was performed. The samples were treated with 30, 50, 70, 85, 95, 100% ethanol/water in sequence for 10 min each. Then, the samples were submitted to 50% hexamethyldisilazane (HMDS)/ethanol mixture and 100% HMDS for 15 min each. The samples were finally dried in the air and coated with a 7 nm thick platinum layer before imaging.

Immunostaining was also used to visualize cell morphologies and cell spreading on the nanoporous and non-porous 3D scaffolds. After 1 day’s cultivation of Hep G2 cells (1 × 10^6^ cells per mL) on the 3D scaffolds, the samples were fixed with paraformaldehyde (4%, PFA, Carl Roth, No. 0335.2) for 2 h. The cells were washed with PBS (1X, Gibco™) three times and permeabilized by Trixon^TM^ X-100 (0.5%, Sigma-Aldrich, T9284) at room temperature for 1 h. Then the samples were placed in BSA (1%, Thermo Scientific™, 37525) blocking solution for 2 h after washing with PBS three times. After removing the blocking solution, the samples were immersed in the solution of Phalloidin-atto 565 (400 nM, Sigma-Aldrich, 94072) for 1 h and DAPI (10 µg mL^−1^, Sigma-Aldrich, MBD0015) for 20 min. After staining, the samples were placed in an ibidi® chamber (200 µL PBS) and imaged by a confocal microscopy (Zeiss LSM 800).

To check the influence of non-specific protein adsorption on cell adhesion, serum-free medium was also used for cell culture on the 3D scaffolds. Here, Hep G2 cells (1 × 10^6^ cells per mL) seeded on the nanoporous and non-porous 3D scaffolds were cultured in Dulbecco’s modified Eagle’s medium (DMEM, Gibco™, and 41966029) without fetal bovine serum (FBS) in a standard incubator (5% CO_2_, 37 °C, Thermo Scientific™). After 1 day culturing, the cells were stained with Calcein-AM (Invitrogen™, 2 µg mL^−1^) to check the coverage of live cells on the 3D scaffolds. The samples were washed with PBS (1X, Gibco™) three times before imaging by a confocal microscope (Zeiss LSM 800). Cell coverage (area) was calculated from the integrated 3D images within a volume of 3 × 3 × 0.3 mm^3^ using the method described above.

### Statistical analysis

Microsoft Office 2013 was used for data statistics and statistical significance calculation. Statistical analysis was performed using Student’s *t* test with statistical significance assigned at **p < 0.01 (moderately significant) and ***p < 0.001 (highly significant).

### Reporting summary

Further information on research design is available in the Nature Research Reporting Summary linked to this article.

## Supplementary information

Supplementary Information

Reporting Summary

## Data Availability

The data that support the findings of this study are available from the corresponding author upon reasonable request. Source data are provided with this paper.

## Source data

Source Data
