# Supplementary material for: 3D printing of inherently nanoporous polymers via polymerization-induced phase separation
Source: Nat Commun. 2021 Jan 11;12:247. doi: 10.1038/s41467-020-20498-1 (PMC7801408; doi:10.1038/s41467-020-20498-1)
Supplement: Supplementary file 1 — Supplementary Information [file 41467_2020_20498_MOESM1_ESM.pdf]

Supplementary Information for

3D printing of inherently nanoporous polymers via polymerization-induced phase separation

Zheqin Dong[1], Haijun Cui[1], Haodong Zhang[2], Fei Wang[2], Xiang Zhan[3], Frederik Mayer[4], Britta Nestler[2], Martin Wegener[4], Pavel A. Levkin[1]*

1. Institute of Biological and Chemical Systems-Functional Molecular Systems (IBCS-FMS) Karlsruhe Institute of Technology, 76344 Eggenstein-Leopoldshafen, Germany
2. Institute of Applied Materials - Computational Materials Science (IAM-CMS), Karlsruhe Institute of Technology, 76131 Karlsruhe, Germany
3. Institute of Micro Process Engineering (IMVT), Karlsruhe Institute of Technology, 76344 Eggenstein-Leopoldshafen, Germany
4. Institute of Nanotechnology and Institute of Applied Physics, Karlsruhe Institute of Technology 76128 Karlsruhe, Germany

*email: levkin@kit.edu

Supplementary Note 1. Ink compositions and printing settings used in DLP 3D printing

The compositions of the inks used in this study are listed as follows:

Ink Mix-1: 30 wt% HEMA, 20 wt% EDMA, 50 wt% cyclohexanol and 4 wt% Irgacure 819 with respects to monomers.

Ink Mix-2: 30 wt% HEMA, 20 wt% EDMA, 40 wt% cyclohexanol, 10 wt% 1-decanol and 4 wt% Irgacure 819 with respects to monomers

Ink Mix-3: 30 wt% HEMA, 20 wt% EDMA, 25 wt% cyclohexanol, 25 wt% 1-decanol and 4 wt% Irgacure 819 with respects to monomers

Ink Mix-4: 30 wt% HEMA, 20 wt% EDMA, 10 wt% cyclohexanol, 40 wt% 1-decanol and 4 wt% Irgacure 819 with respects to monomers

Ink Mix-5: 30 wt% HEMA, 20 wt% EDMA, 50 wt% 1-decanol and 4 wt% Irgacure 819 with respects to monomers

Ink Mix-6: 60 wt% HEMA, 40 wt% EDMA and 4wt% Irgacure 819 with respects to monomers

Ink Mix-7: 30 wt% DMAEA, 20 wt% EDMA, 40 wt% cyclohexanol, 10 wt% 1-decanol and 4 wt% Irgacure 819 with respects to monomers

Ink Mix-8: 60 wt% DMAEA, 40 wt% EDMA and 4 wt% Irgacure 819 with respects to monomers

Printing settings: In DLP 3D printing, the layer thickness and the layer cure time are two important parameters that directly influence the printing speed and quality. In principle, the choice of layer thickness is only limited by the translation precision of the platform. However, there is always a trade-off between printing speed and resolution when adjusting the layer thickness. The layer cure time, on the other hand, needs to be judiciously selected: too short cure time will lead to layer delamination or missing parts, while too long cure time will result in overgrowth and lamination issues between the specimen and vat lining.

Based on these considerations, the layer thickness for all 3D printing experiments in this study was selected as 50 μm to ensure good printing resolution with acceptable printing speed. For determining the cure time, the working curve (cure depth as a function of cure time) for each ink was first

measured (Supplementary Fig. 6b and Supplementary Fig. 10). The working curve provides a reference to determine the cure time. Then the cure time was empirically determined to provide objects with visibly good printability, without overgrowth, missing parts, or lamination issues with vat lining. The cure time was set as 12.5 s for ink Mix-1 to Mix-6, and 25 s for ink Mix-7 and Mix-8 due to the lower curing rate of DMAEA.

Supplementary Note 2. UV-Vis spectrum of the inks prepared with different photoinitiators

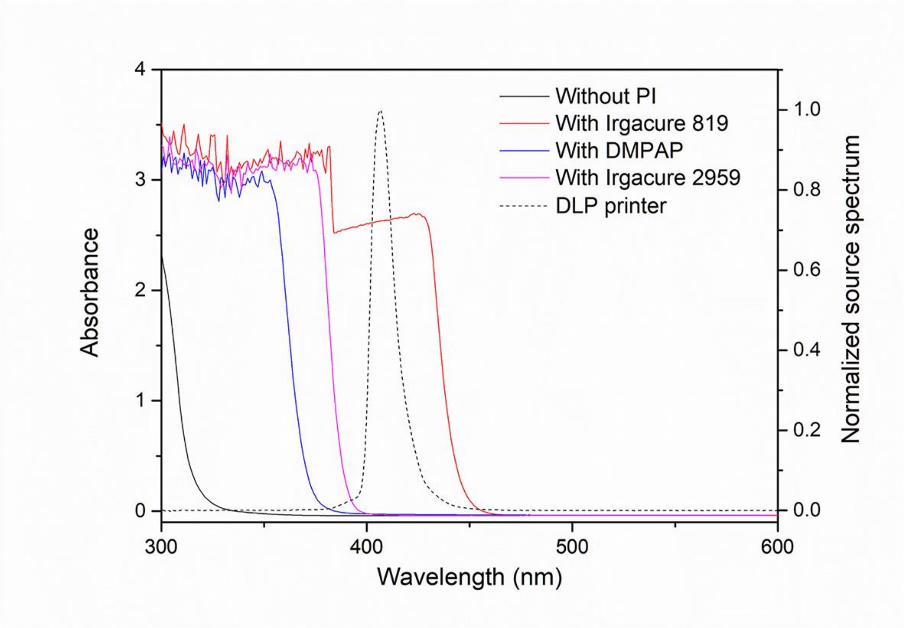

Supplementary Figure 1. UV-Vis spectra of the inks (30 wt% HEMA, 20 wt% EDMA, 40 wt% cyclohexanol and 10 wt% 1-decanol) without photoinitiators and with different photoinitiators (4 wt% to monomers), compared with the emission spectrum of the DLP printer.

Supplementary Note 3. Optimization of the ink composition

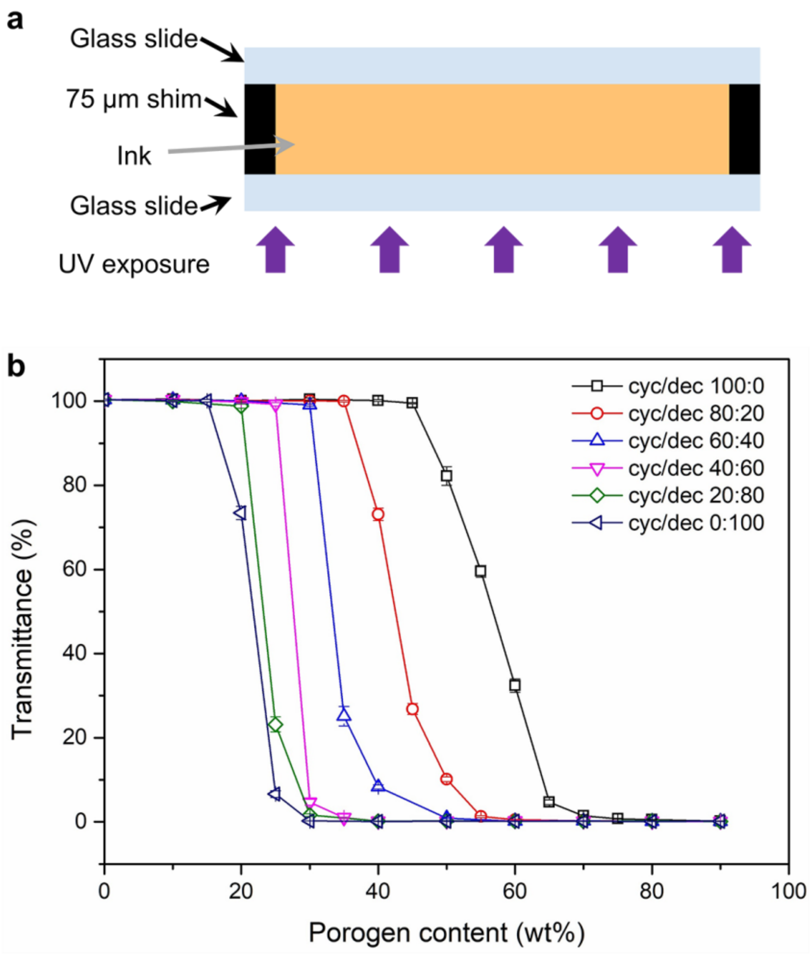

Supplementary Figure 2. a, Schematic showing the fabrication of 75 μm thick films by photopolymerization (300 s UV irradiation). **b**, Transmittance of the films prepared using inks with a range of monomer/porogen compositions at 500 nm. The ratio of HEMA/EDMA in the monomer is fixed at 3:2 (w/w) for all inks. Error bars are standard deviations of three independent experiments (N=3).

To realize the 3D printing of inherently nanoporous polymers, the ink should undergo phase separation upon photopolymerization. Therefore, we screened inks with a range of monomer and porogen ratios and identified the compositions that could undergo phase separation upon photopolymerization. Specifically, we irradiated the ink with the UV light source of the 3D printer for 300 s to prepare a 75 μm thick film and measured its transmittance at 500 nm (Supplementary Fig. 2a). At a fixed porogen composition, when the porogen content in the inks exceeded a certain

value, the light transmittance of the dry film experienced a dramatic decrease (Supplementary Fig. 2b), indicating phase separation in the photopolymerization process. This method was used to construct the ternary diagram shown in Fig. 1c.

Another criterion in selecting the ink composition is the mechanical strength of the 3D printed structures. We found that the compressive strength of the 3D printed structures decreased with increasing the porogen content in the ink due to a decrease in their density (Supplementary Fig. 3) When the porogen content was higher than 80 wt%, the mechanical strength of the 3D printed structures was too weak leading to a spontaneous degradation of the structures during 3D printing.

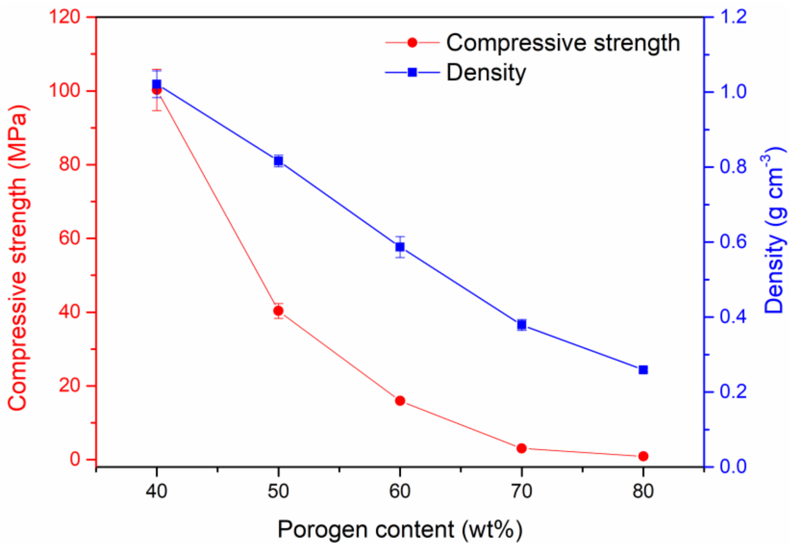

Supplementary Figure 3. Compressive strength and density of 3D printed polymer cubes (5×5×5 mm^3) printed using inks with different porogen content. The ratio of HEMA/EDMA and cyclohexanol/1-decanol in the inks is fixed at 3:2 (w/w) and 4:1 (w/w), respectively. Error bars are standard deviations of three independent experiments (*N*=3).

Supplementary Note 4. Investigation of the printing resolution

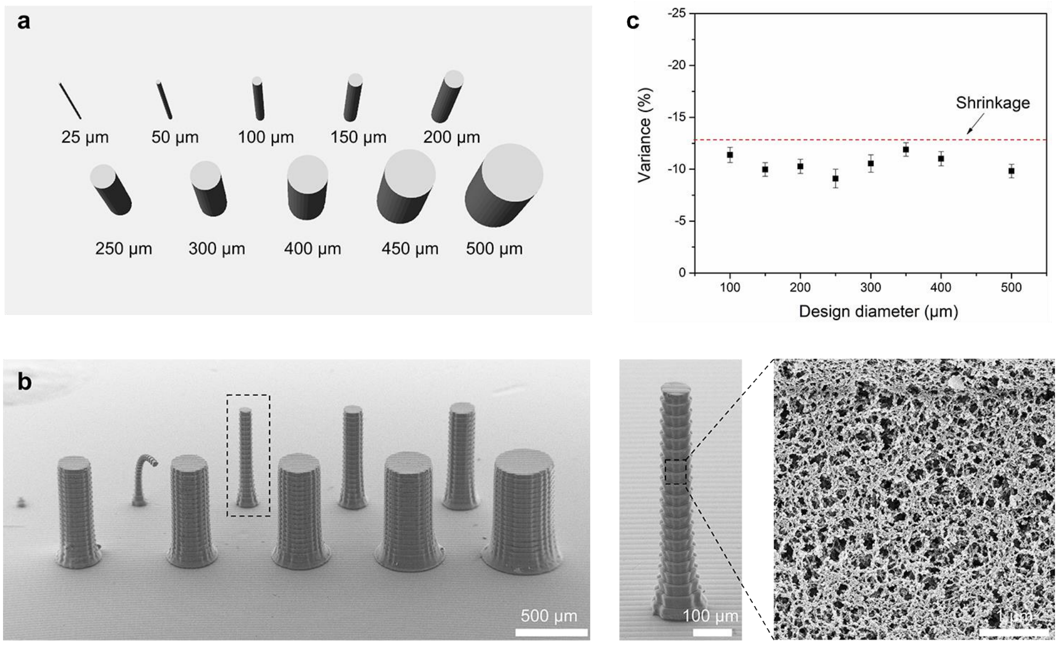

Supplementary Figure 4. a, 3D design of an array of pillars with different diameters (height 1 mm). **b**, SEM micrographs of the 3D printed pillar array. **c**, Measured deviation between the designed and printed pillar diameter. Error bars are standard deviations of three independent experiments (*N*=3).

For investigation of the printing resolution, we used a DLP printer with a higher resolution (Miicraft prime 110, pixel size 40 µm, https://miicraft.com/). An array of pillars with different diameters was designed and printed (Supplementary Fig. 4a). It can be seen from the SEM images that pillars with diameter larger than 100 µm can be printed, and the surface of the pillars remained highly porous (Supplementary Fig. 4b). The variance between the designed and printed feature was calculated by measuring the diameter of the printed pillars shown in the SEM images. All printed pillars showed -8~13% variance compared to the designed value (Supplementary Fig. 4c). This is attributed to the shrinkage (~13%) of the printed porous polymers during supercritical drying, calculated from the length of a 3D printed cube (5×5×5 mm^3) before and after drying using optical microscopy. We note that there is a slight difference between the geometry of the printed pillars and the CAD files: the printed pillars are slightly wider at the bottom. This might be attributed to the higher crosslinking density (potentially due to the reflection of the substrate or the effect of the surface on the composition of the porogens) at the bottom and thus a lower shrinkage in the drying process.

Supplementary Note 5. Mechanical properties of the 3D printed non-porous, nanoporous and hierarchically porous structures

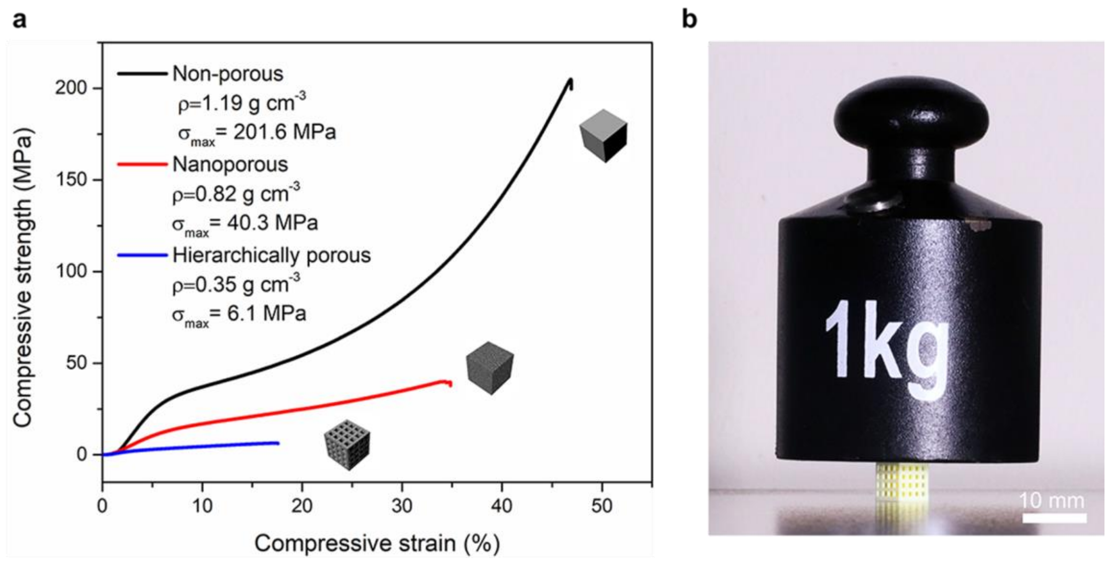

Supplementary Figure 5. a, Compressive strength-strain curves for a non-porous polymer cube (5×5×5 mm^3) printed using an ink without porogens (Mix-6), a nanoporous polymer cube (5×5×5 mm^3) and a hierarchically porous polymer cube (5×5×5 mm^3: pore size 0.675 mm, wall thickness 0.45 mm, macroscopic porosity 43%) printed using an ink with porogens (Mix-2). Polymer: poly(HEMA-co-EDMA). Representative of three independent experiments (*N*=3). **b**, A photograph showing the hierarchically porous cube supporting a weight of 1 kg.

Supplementary Note 6. Viscosity and reactivity of the five inks with different porogen compositions

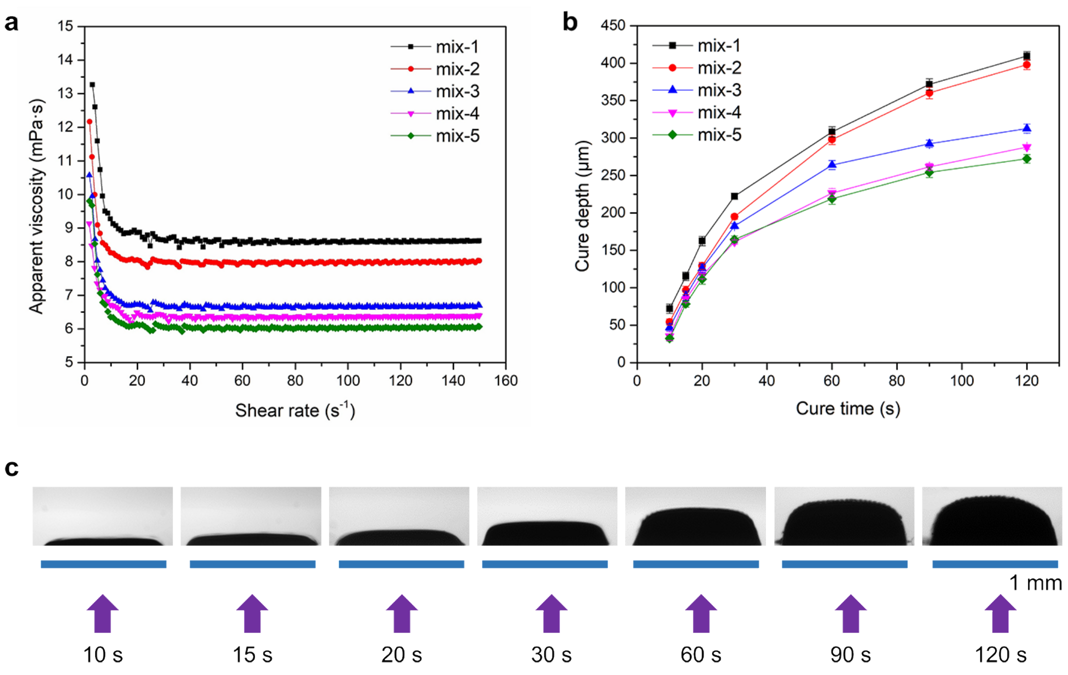

Supplementary Figure 6. a, Apparent viscosity as a function of shear rate for the indicated five inks with different porogen compositions. **b**, Cure depth as a function of cure time for the indicated fives inks. Error bars are standard deviations of three independent experiments (*N*=3). **c**, Cure depth determination: a 1×1 mm^2 square was irradiated by the UV light source of the 3D printer for different periods of time, and then the height of cured structures (black in the pictures) was measured.

Supplementary Note 7. Theoretical simulation of polymerization-induced phase separation using the phase-field method

A phase-field model within the framework of the Cahn-Hilliard approach is adopted to simulate the polymerization-induced phase separation. In our model, we use c_1, c_2, c_3 to depict the concentrations of monomer, polymer, and solvent, respectively. The time evolution of the respective concentration c_i follows[1]:

$$\frac{\partial c_i}{\partial t} = \nabla \cdot \left(\sum_{j=1}^{3} M_{ij} \nabla \frac{\delta F}{\delta c_j} \right). \quad (1)$$

In Supplementary Equation 1, M_{ij} is the mobility representing the kinetic parameter of the system and assigned in accordance with the Onsager relationship as

$$M = D_0 \begin{bmatrix} c_1(1-c_1) & -c_1c_2 & -c_1c_3 \\ -c_1c_2 & c_2(1-c_2) & -c_2c_3 \\ -c_1c_3 & -c_2c_3 & c_3(1-c_3) \end{bmatrix}.$$

Here, D_0 stands for the inter-diffusivity of the polymer solution and is formulated as $\sum_{i=1}^{3} D_i c_i$, in which D_i is the self-diffusivity for component i. The symbol δ denotes the variational derivative. F represents the free energy functional of the system scaled by k_bT (k_b - Boltzmann constant, T - temperature) and reads

$$F = \int_{\Omega} \left[f(c_1, c_2, c_3) + \sum_{i=1}^{3} \kappa_i (\nabla c_i)^2 \right] d\Omega \quad (2)$$

where κ_i is the gradient energy coefficient for component i and Ω denotes the domain occupied by the system. Pertaining to the Flory-Huggins theory, the bulk free energy density $f(c_1, c_2, c_3)$ is expressed as[2]

$$f = T(\frac{c_1 \ln c_1}{N_1} + \frac{c_2 \ln c_2}{N_2} + c_3 \ln c_3) + \chi_{12}c_1c_2 + \chi_{13}c_1c_3 + \chi_{23}c_2c_3 + \chi_{123}c_1c_2c_3 \quad (3)$$

The parameter N_1 represents the degree of polymerization (DP) of the monomers (N_1=1), N_2 denotes the average DP of the polymers (N_2=20). The Flory parameters $\chi_{12}, \chi_{13}, \chi_{23}$ recount the interaction between the monomers and the polymer, the monomers and the porogen, and the polymer and the porogen, respectively. The parameter χ_{123} depicts the ternary interaction of the monomers, polymer, and porogens. A positive χ describes a repulsive intermolecular force in the poor-solvent system and a negative χ narrates an attractive force. Due to the paucity of the experimental data for the Flory parameters, the following assumptions are made. (i) $\chi_{12} = 0.1$ gives the good

miscibility of monomer in polymer. (ii) $\chi_{13} = 0.5$ demonstrates the lower miscibility of monomer in the porogens compared with its solubility in polymer. (iii) χ_{23} is postulated to increase with the 1-decanol concentration in the inks (Supplementary Table 1). As 1-decanol is a poorer solvent than cyclohexanol, increasing 1-decanol concentration leads to a poorer solvent system, giving rise to the larger repulsive interactions between solvent and polymer. The last parameter χ_{123} is assigned as -3.0 to simulate the polymerization-induced phase separation.

Supplementary Equation 1 coupling with Supplementary Equation 3 is numerically solved by the finite difference method. A parallelization of the numerical algorithm is achieved with Message Passing Interface (MPI) techniques. The simulation results are visualized by the software Paraview.

Supplementary Table 1. Ink mixtures used for producing inherently nanoporous 3D polymers and the Flory parameter χ_{23} used for calculating the free energy density

Mix Number	HEMA (wt%)	EDMA (wt%)	1-Decanol (wt%)	Cyclohexanol (wt%)	χ_{23}
1	30 wt%	20 wt%	0 wt%	50 wt%	2.0
2	30 wt%	20 wt%	10 wt%	40 wt%	2.5
3	30 wt%	20 wt%	25 wt%	25 wt%	3.0
4	30 wt%	20 wt%	40 wt%	10 wt%	4.0
5	30 wt%	20 wt%	50 wt%	0 wt%	5.0

Supplementary Note 8. Physical properties of the 3D structures printed using the five inks with different porogen compositions

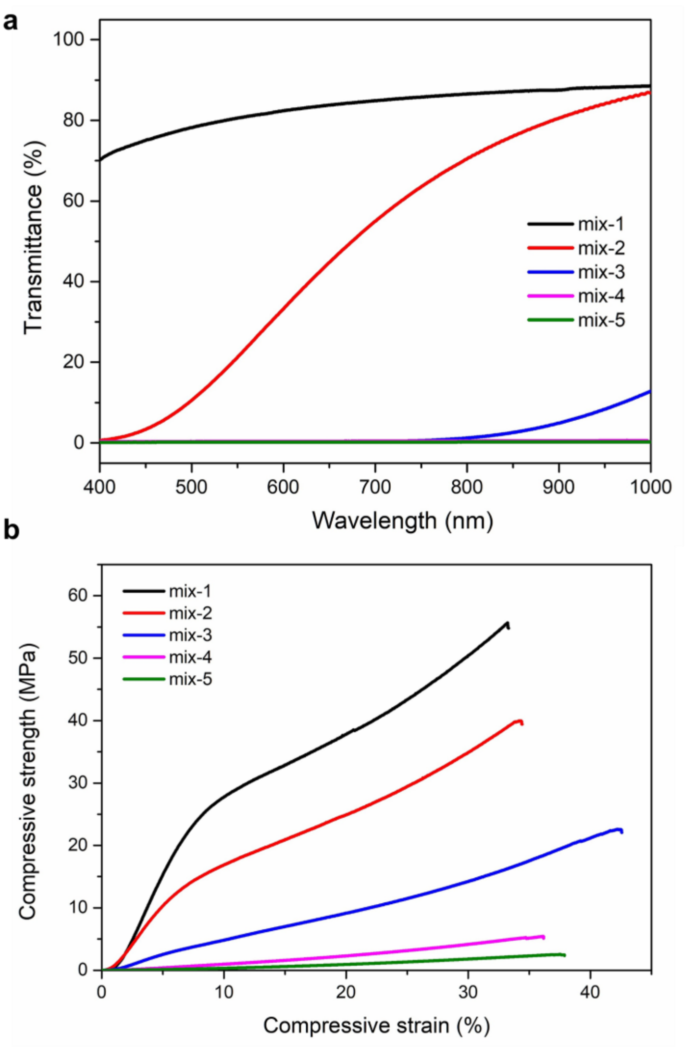

Supplementary Figure 7. a, UV-Vis transmittance of 75 μm films printed using the indicated five inks with different porogen compositions. Representative of three independent experiments (N=3). **b**, Compressive strain-curve for 3D polymer cubes (5×5×5 mm^3) printed using the five inks. Representative of three independent experiments (N=3).

Supplementary Note 9. Pore size distribution of the 3D printed object with tri-disperse pore size

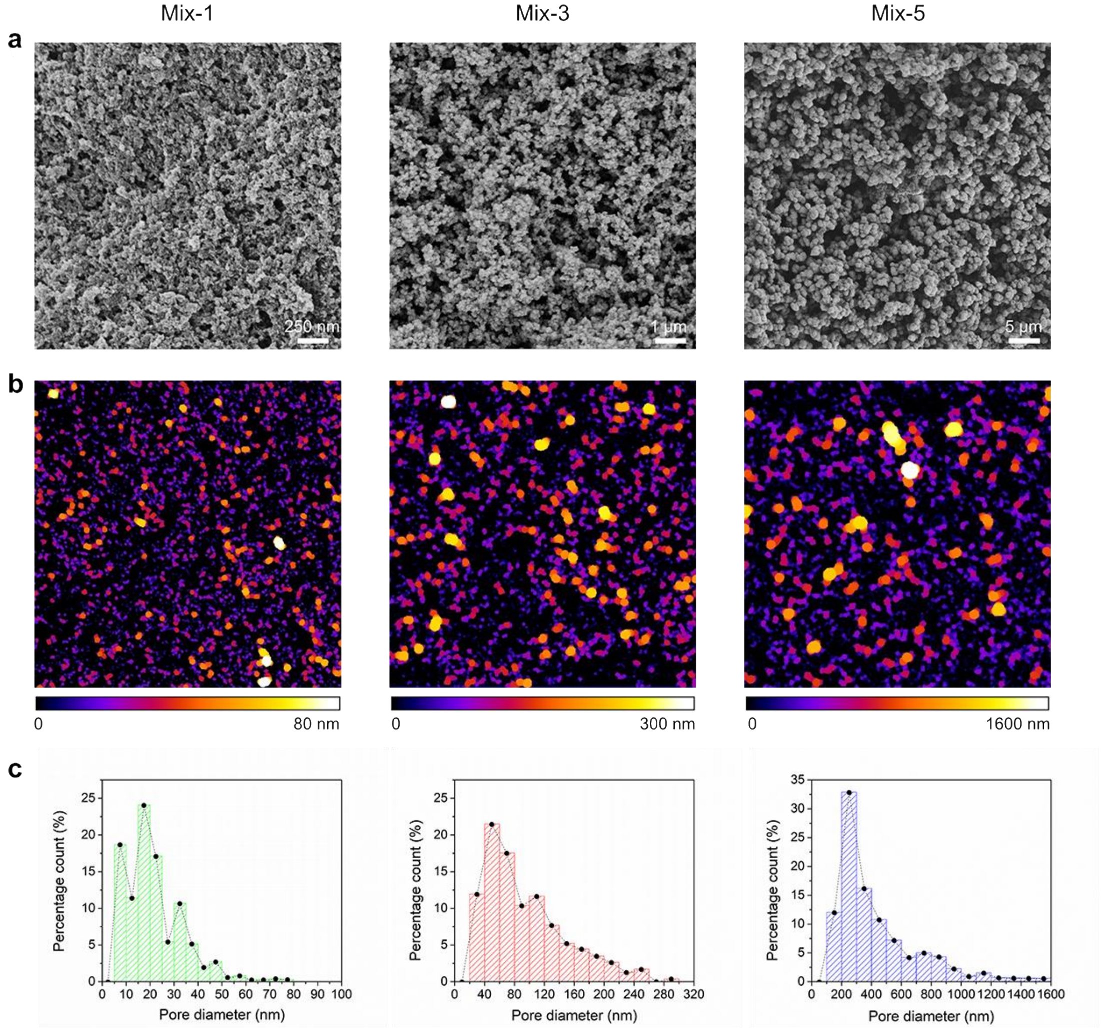

Supplementary Figure 8. Pore size distribution of the 3D printed object with tri-disperse porosity by switching the inks during printing (Fig. 3f). **a**, Cross-sectional SEM images; **b**, Local thickness mapping; **c,** Pore size distribution.

The cross-sectional SEM images were taken at the upper-part, middle-part and lower-part of the 3D printed object (Fig. 3f), which were printed by using ink Mix-1, Mix-3 and Mix-5, respectively (Supplementary Fig. 8a). The pore sizes were measured from the cross-sectional SEM images using the 'Local Thickness' plug-in for ImageJ. The SEM images were first converted to binary images using greyscale thresholding. Then the binary images were analyzed using the 'Local Thickness' plug-in, which measures the diameter of the largest sphere that fits into the dark region (pores), giving back a colored map of local thickness (Supplementary Fig. 8b). Here we assume that the

pores preserve a spherical shape and the measured local thickness represents the pore diameter. The histograms for pore size distribution were calculated from the pixel counts for a given thickness (Supplementary Fig. 8c). We note that the extraction of pore size from 2D cross-sectional instead of 3D z-stack images may lead to a systematic error, which we do not correct as here we care more about the general trends rather than very exact numbers for the pore diameter.

Supplementary Note 10. Mechanical properties of the 3D printed heterogeneous structures

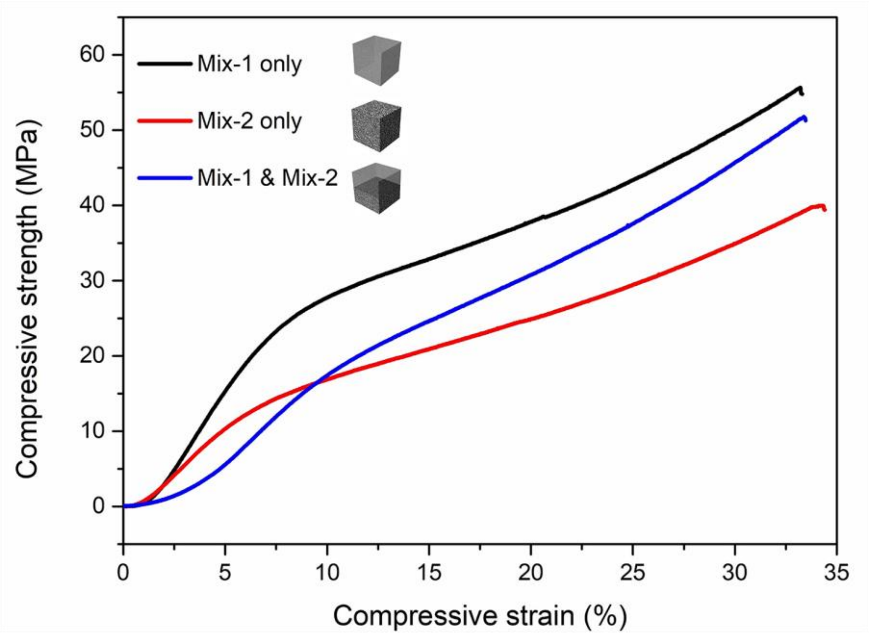

Supplementary Figure 9. Compressive strain-curve for 3D printed polymer cubes (5×5×5 mm^3), one printed using ink Mix-1 only, one printed using ink Mix-2 only, and one printed using ink Mix-1 for the upper half part and ink Mix-2 for the lower half part. Representative of three independent experiments (*N*=3).

Supplementary Note 11. Curing depth as function of cure time for inks using dimethyl aminoethyl methacrylate as the monofunctional monomer

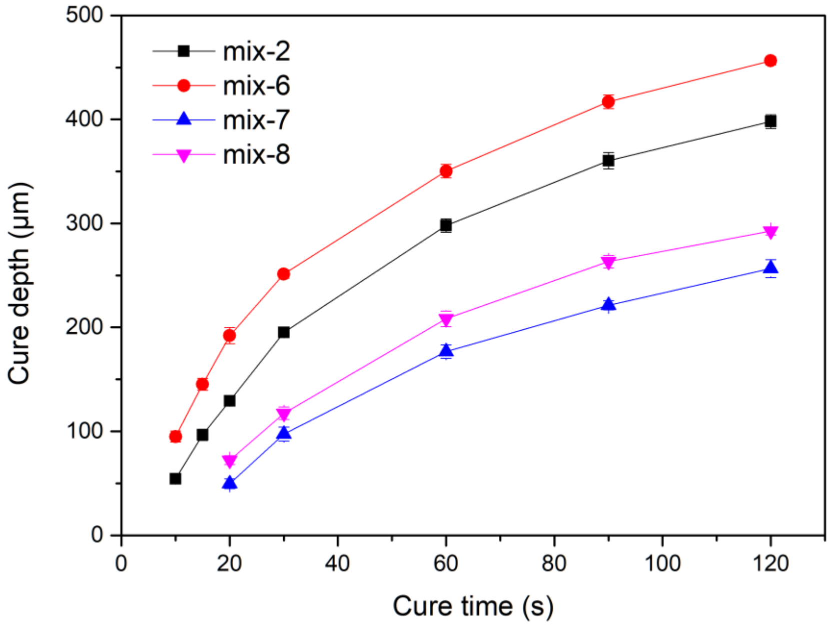

Supplementary Figure 10. Curing depth as a function of cure time for inks using dimethyl aminoethyl methacrylate (DMAEA) as the monofunctional monomer (Mix-7, with porogens; Mix-8, without porogens), compared to inks using hydroxyethyl methacrylate (HEMA) as the monofunctional monomer (Mix-2, with porogens; Mix-6, without porogens). Error bars are standard deviations of three independent experiments (*N*=3).

Supplementary Note 12. Microstructure of 3D printed cubes using dimethyl aminoethyl methacrylate as the monofunctional monomer

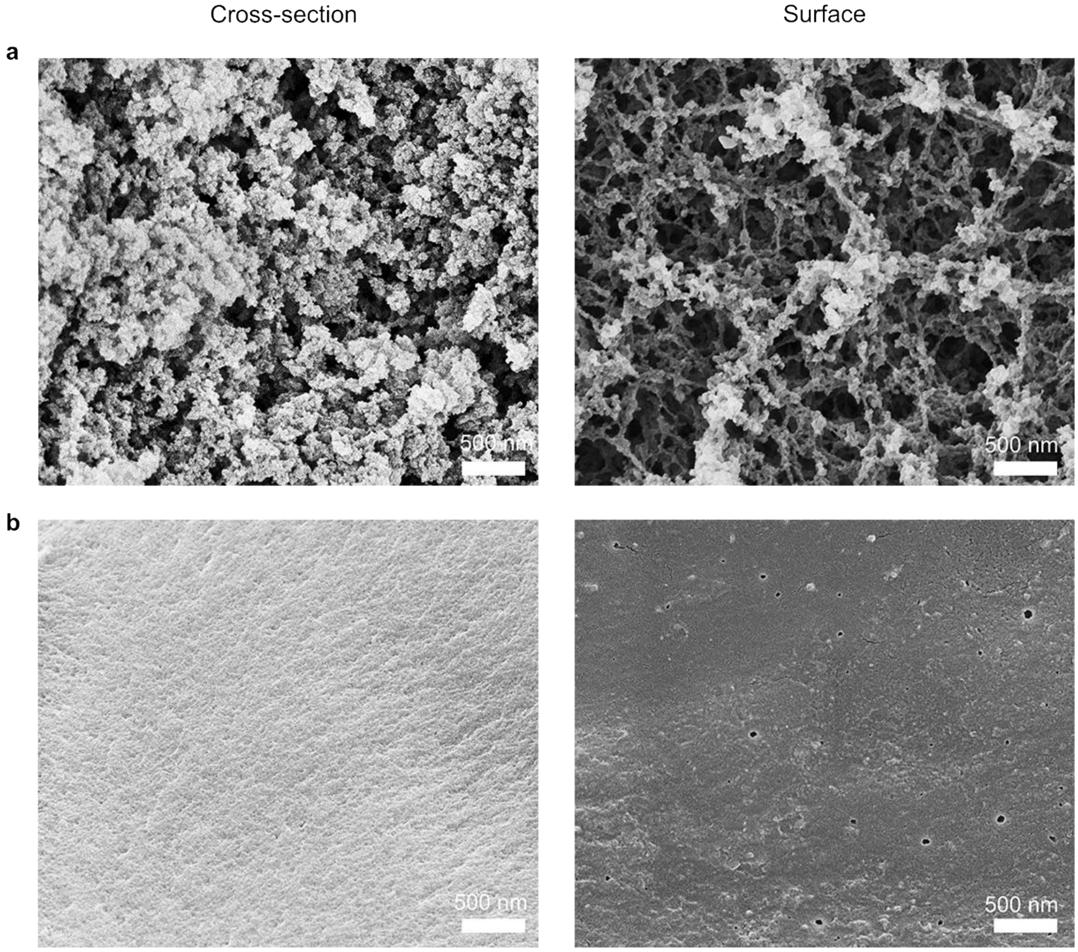

Supplementary Figure 11. SEM micrographs of solid cubes (5×5×5 mm^3) printed using dimethyl aminoethyl methacrylate as the monofunctional monomer: **a,** an inherently nanoporous cube printed using an ink with porogens (Mix-7) and **b,** a non-porous cube printed using an ink without porogens (Mix-8).

Supplementary Note 13. Adsorption kinetics of the 3D printed non-porous, nanoporous and hierarchically porous adsorbents

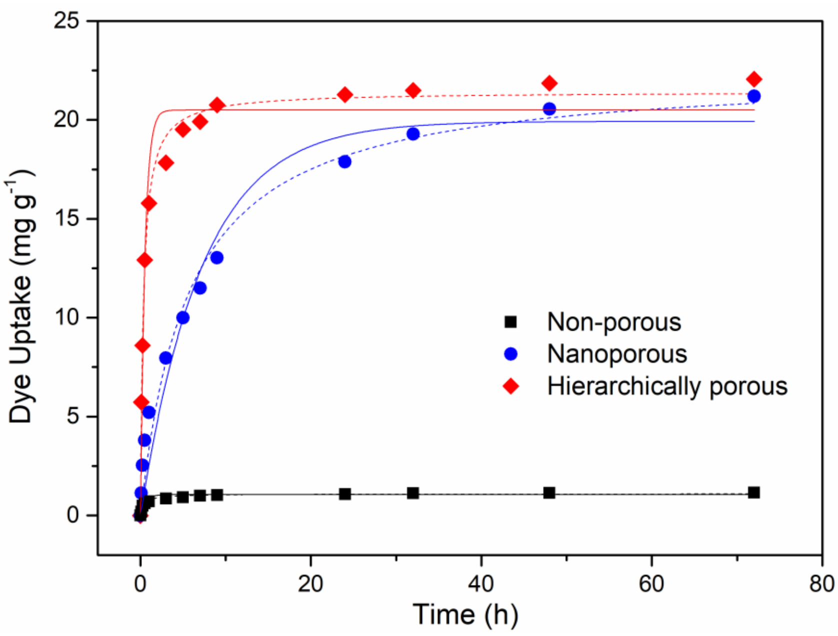

Supplementary Figure 12. Time-dependent dye uptake by the 3D printed non-porous, nanoporous and hierarchically porous adsorbents in 72 hours, with pseudo-first order kinetic modeling (solid lines) and pseudo-second order modeling (dash lines).

Supplementary Table 2. Summary of the adsorption kinetic modeling parameters from Supplementary Figure 12. q_e, K_1, K_2 are the parameters estimated by fitting, and represent equilibrium uptake, first and second order kinetic constants, respectively.

	Pseudo-first order			Pseudo-second order		
	R^2<br>-	q_e<br>(mg g^{-1})	K_1<br>(h^{-1})	R^2<br>-	q_e<br>(mg g^{-1})	K_2<br>(g mg^{-1} h^{-1})
Non-Porous	0.92	1.03	1.70	0.97	1.09	2.07
Nanoporous	0.96	18.90	0.15	0.98	21.00	0.0099
Hierarchically porous	0.98	19.19	2.28	0.99	19.92	0.18

Pseudo-first order kinetic model (Lagergren model): This model assumes that the pollutant uptake (*q*) increase with time (*dq*/*dt*) is proportional to the difference between *q* and the uptake at equilibrium (q_e)[3].

$$\frac{dq}{dt} = K_1(q_e - q) \tag{4}$$

$$q = q_e\,(1 - e^{-K_1 t}) \tag{5}$$

Pseudo-second order kinetic model (Ho model): This model assumes that the pollutant uptake (q) increase with time (*dq*/*dt*) is proportional to the difference between *q* and the uptake at equilibrium (q_e) to the second power[4].

$$\frac{dq}{dt} = K_2(q_e - q)^2 \tag{6}$$

$$q = \frac{K_2 {q_e}^2 t}{1 + K_2 q_e t} \tag{7}$$

Supplementary Note 14. Cell viability on the nanoporous and non-porous 3D scaffolds

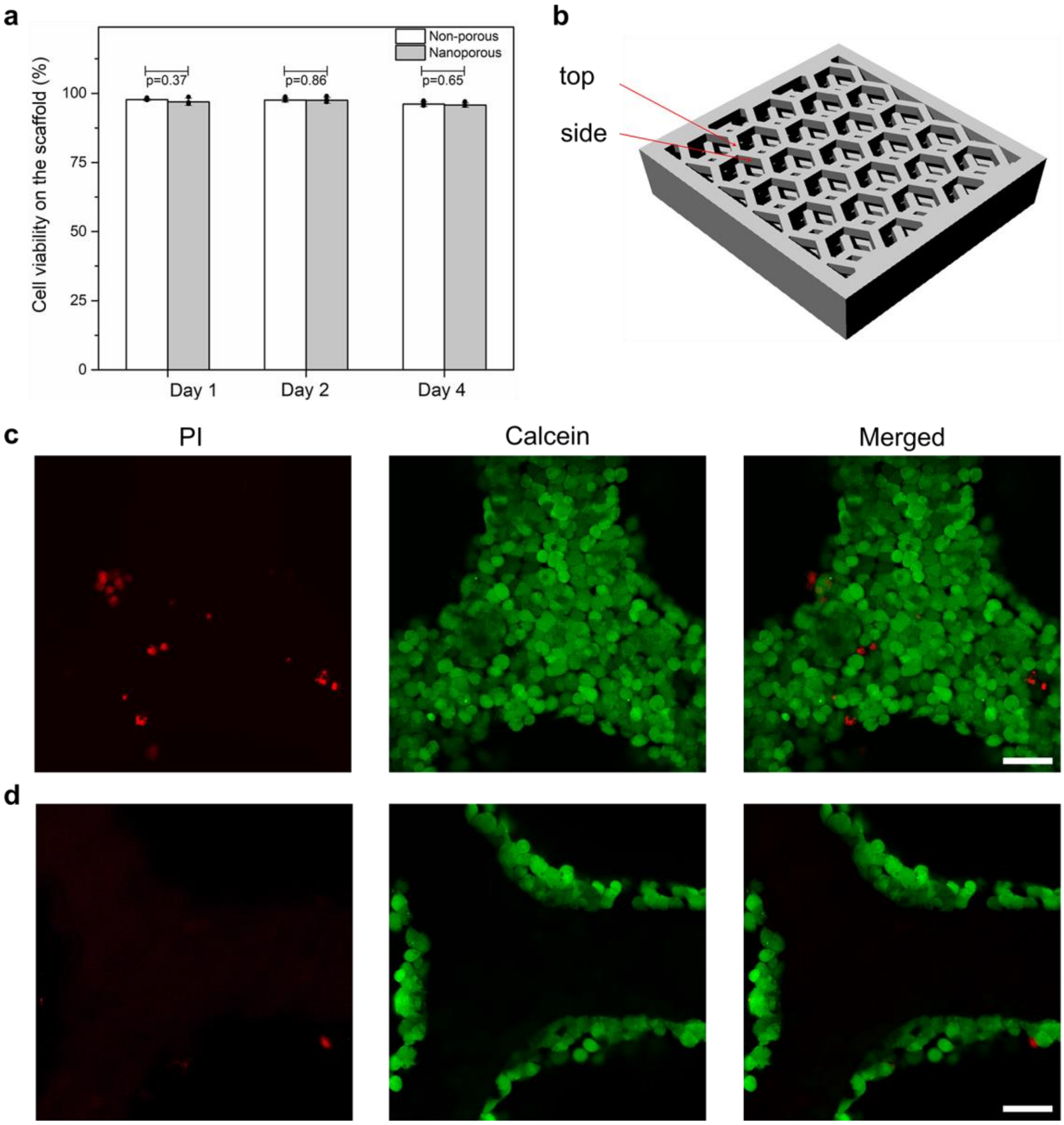

Supplementary Figure 13. a, Cell viability (based on Calcein/PI staining) on the inherently nanoporous 3D scaffold and the non-porous 3D scaffold after 1, 2 and 4 days of culture. The scaffolds were washed with PBS three times before imaging. Error bars are standard deviations of three independent experiments (N=3). Five replicates (n=5) were used in each experiment to calculate the cell viability. The statistical significance was assessed using unpaired two-tailed Student’s t test. **b**, Schematic representation of the scaffold´s geometry. **c-d**, Representative 2D confocal images of Hep G2 cells cultured on the inherently nanoporous 3D scaffold after 4 days. **c**, top of the 3D scaffold; **d,** side of the scaffold. Scale bars: 50 µm.

Supplementary Note 15. Cell morphology on the nanoporous and non-porous 3D scaffolds

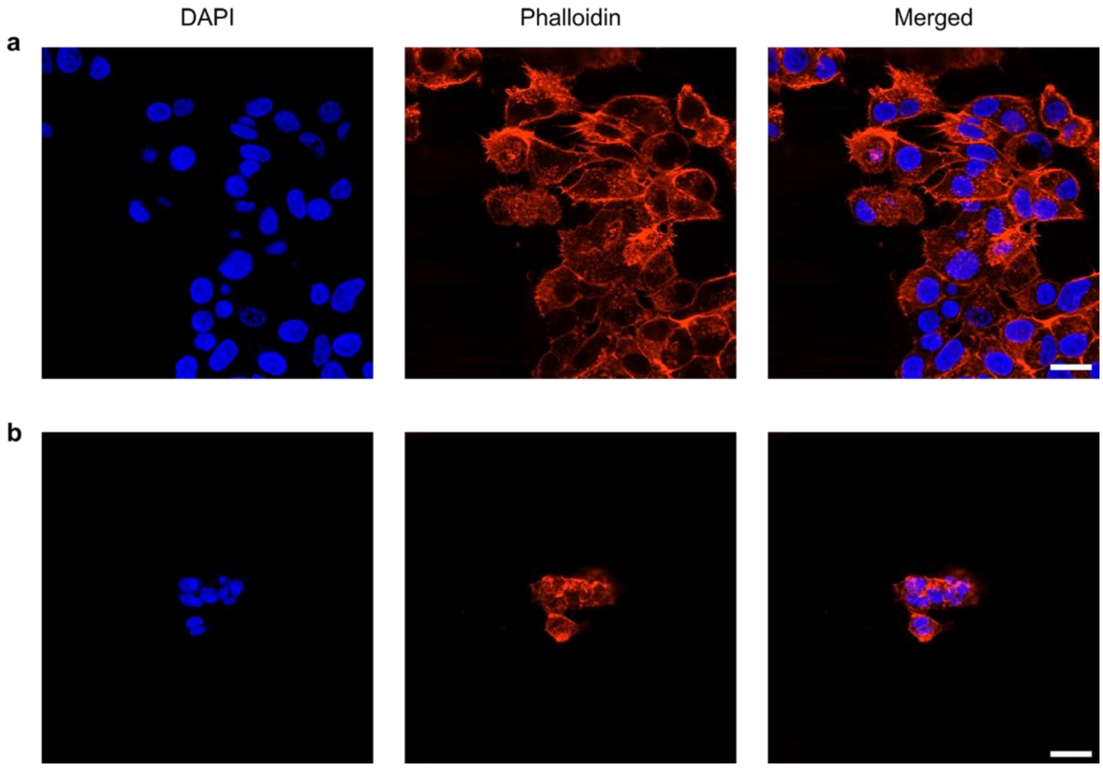

Supplementary Figure 14. 2D confocal microscopy images of Hep G2 cells cultured on the inherently nanoporous 3D scaffold (**a**) and the non-porous 3D scaffold (**b**) after 1 day. In these images, phalloidin stains the fiber actin, DAPI stains the DNA nucleus blue. Scale bars: 20 μm. The microscopy images shown are representative of five replicates (*n*=5) over three independent experiments (*N*=3).

Supplementary Note 16. Cell coverage on the nanoporous and non-porous 3D scaffolds in serum-free medium

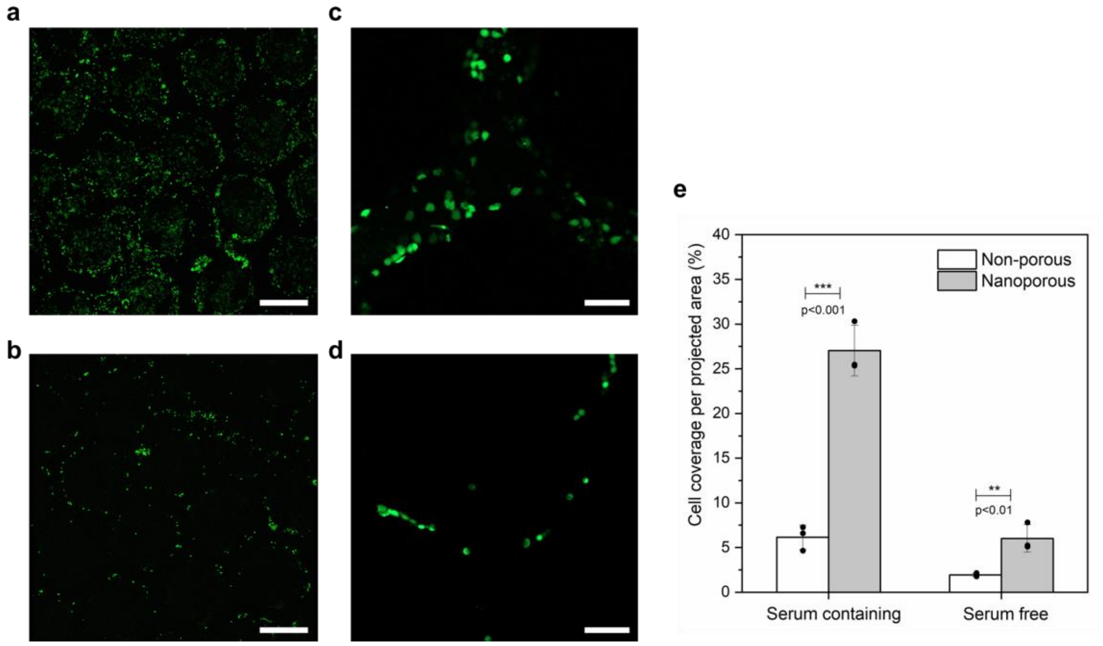

Supplementary Figure 15. a-b, 3D confocal microscopy images of Hep G2 cells cultured on the inherently nanoporous scaffold (**a**) and the non-porous scaffold (**b**) after 1 day of culture in a serum-free medium. Scale bars: left: 500 µm. The 3D confocal images were integrated from 30 z-stack images with a single stack thickness of 10 um. **c-d**, Representative 2D confocal images showing the cell morphology on the nanoporous scaffold (**c**) and the non-porous scaffold (**d**). Scale bars: left: 50 µm. **e,** Coverage of live cells (Calcein-positive) per projected area per projected area calculated from the 3D confocal images within a volume of $3\times3\times0.3$ mm^3. The results were compared with those obtained in a serum-containing medium (Fig. 5d). Error bars are standard deviations of three independent experiments (N=3). The statistical significance was assessed using unpaired two-tailed Student's t test.

Supplementary Note 17. Cell culture on the nanoporous and non-porous 2D plates

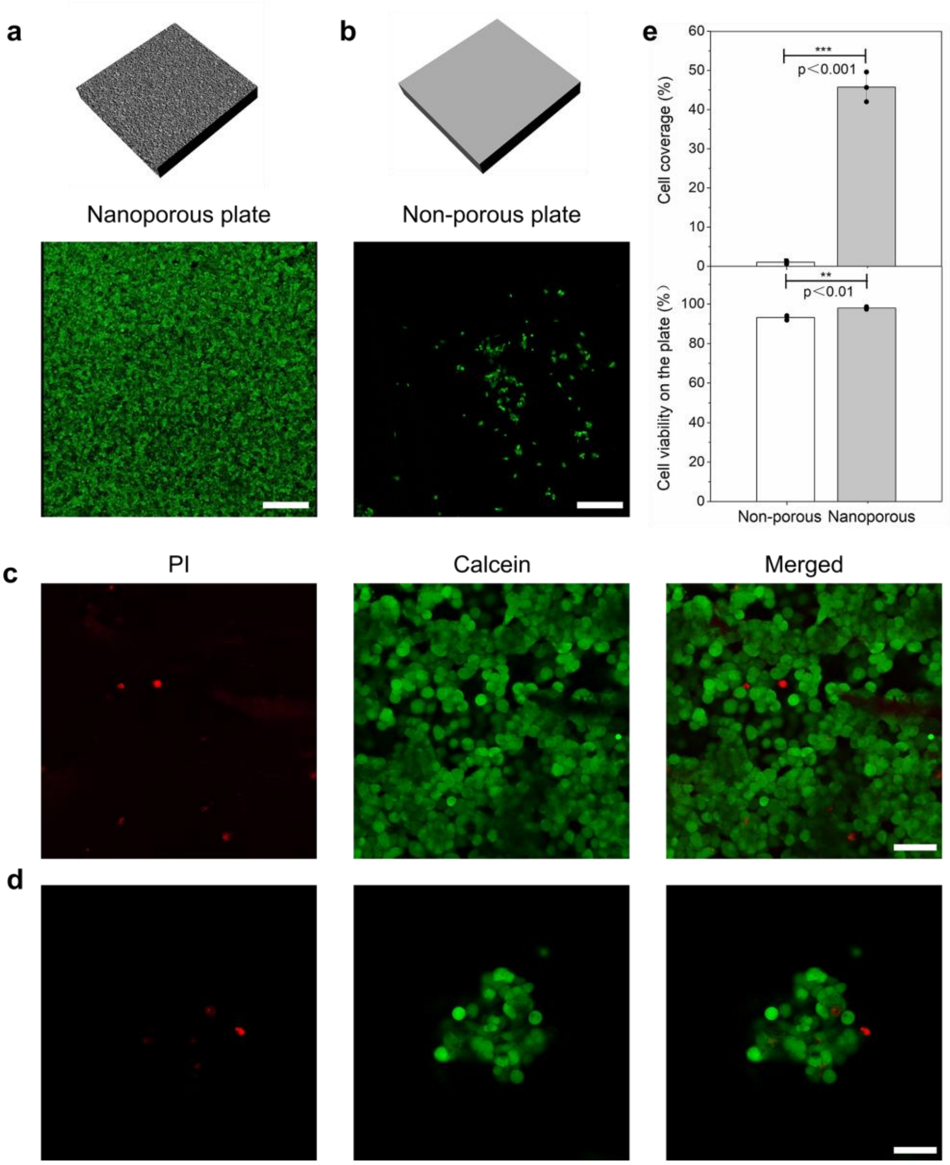

Supplementary Figure 16. a-b, Overview 2D confocal microscopy images of Hep G2 cells cultured on an inherently nanoporous polymer plate printed using Mix-2 (**a**) and a non-porous polymer plate printed using Mix-6 (**b**) after 1 day. Scale bars: 300 μm. **c-d**, Higher magnification images showing cell viability on the nanoporous (**c**) and non-porous (**d**) plates after washing with PBS. Scale bars: 50 μm. **e**, Cell coverage and viability (based on Calcein/PI staining) on the nanoporous plate and non-porous plates after 1 day culturing. Initial cell seeding density was 1×10^6 cells/mL. Cell coverage (based on fluorescence signal) was calculated within an area of 1.8×1.8 mm^2. Error bars are standard deviations of three independent experiments (N=3). Five replicates (n=5) were used in each experiment to calculate the cell viability. The statistical significance was assessed using unpaired two-tailed Student's t test.

Supplementary Note 18. Schematic of the 3D printer setup

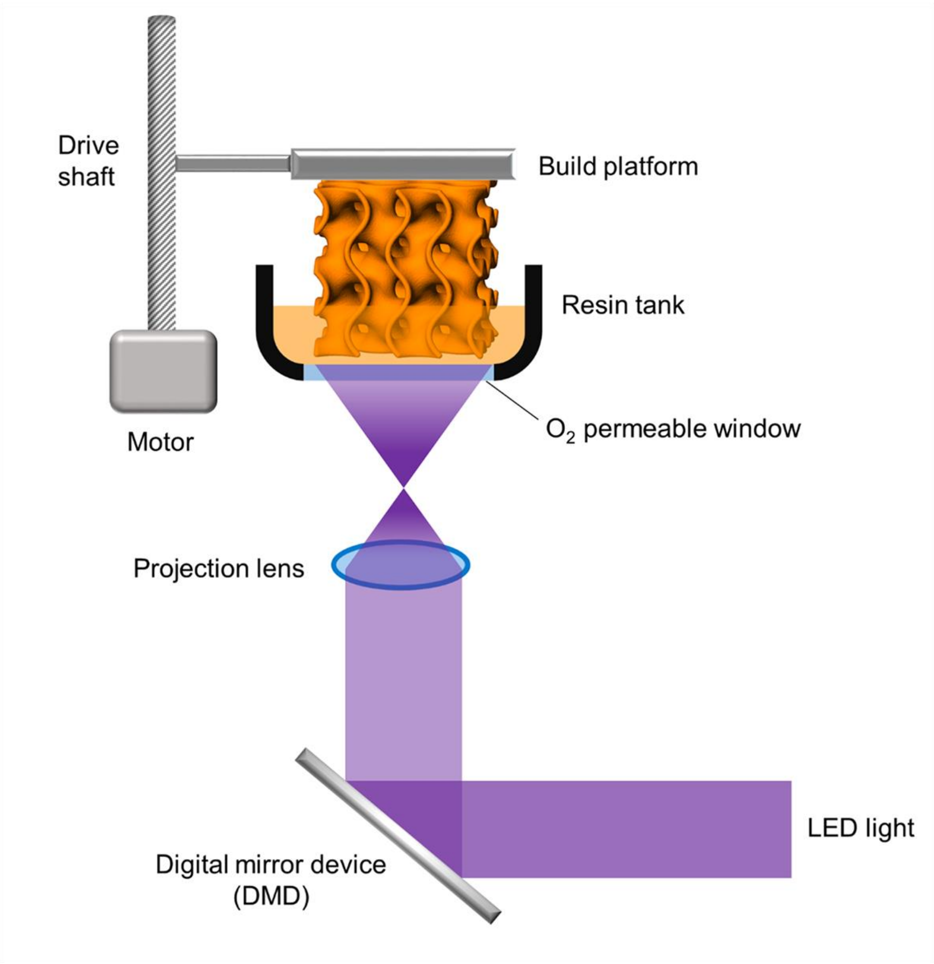

Supplementary Figure 17. Schematic showing the setup of the DLP 3D printer.

Supplementary Note 19. Volume change of the 3D printed nanoporous polymers in acetone

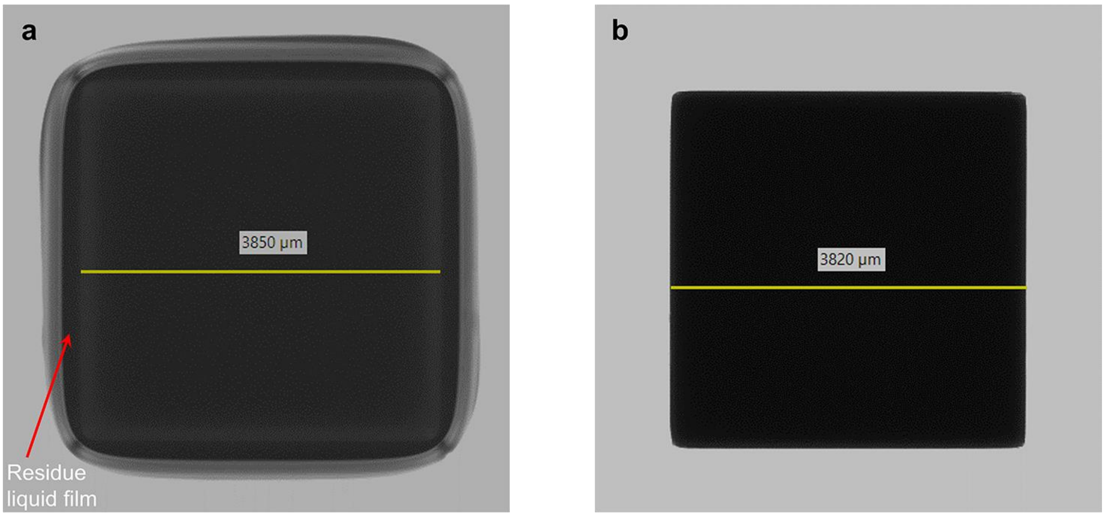

Supplementary Figure 18. Microscopy images of **a**, an as-printed 3D printed cube and **b**, the cube after immersing in acetone for 24 h (without drying). Representative of three independent experiments (N=3).

To check whether acetone would alter the structure of the 3D printed nanoporous polymers, we calculated the volume of an as-printed 3D printed cube and the cube after immersing in acetone for 24 h. The volume change turns out to be very low (<3%), which ensures the structural fidelity of the 3D printed objects.

Supplementary Note 20. Calculation of the BET specific surface area

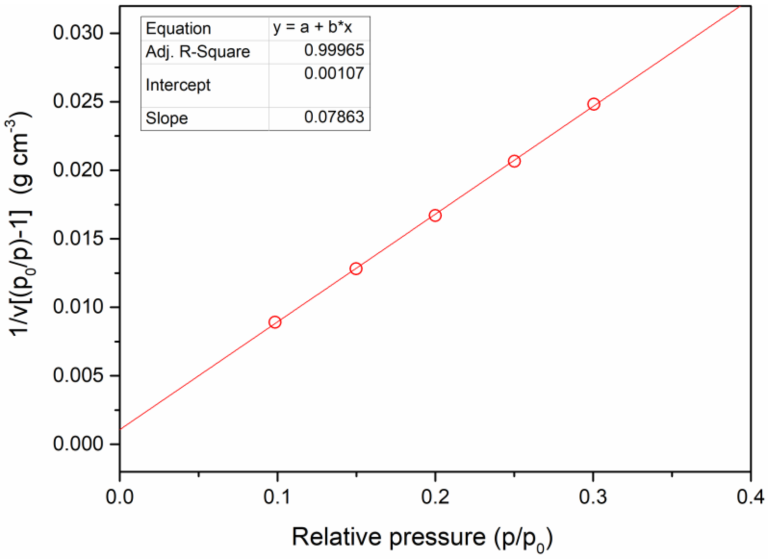

Supplementary Figure 19. A BET plot for 3D nanoporous polymers printed using Mix-2.

The specific surface area was calculated using the Brunauer–Emmett–Teller (BET) equation:

$$\frac{1}{v\left[\left(\frac{p_0}{p}\right)-1\right]} = \frac{c-1}{v_m c}\left(\frac{p}{p_0}\right) + \frac{1}{v_m c} \tag{8}$$

Where p and p_0 are the equilibrium and the saturation pressure of N_2 at the temperature of adsorption, v is the adsorbed gas quantity, and v_m is the monolayer adsorbed gas quantity. c is the BET constant. Supplementary Equation 8 can be plotted as a straight line with $\frac{1}{v\left[\left(\frac{p_0}{p}\right)-1\right]}$ on the y-axis and $\left(\frac{p}{p_0}\right)$ on the x-axis. This plot is called a BET plot. As the linear relationship of this equation is maintained only in the range of $0.05 < \left(\frac{p}{p_0}\right) < 0.35$, five individual points in this range were used to determine the BET plot (Supplementary Fig. 19). The value of the slope a and the y-intercept b of the line were used to calculate the monolayer adsorbed gas quantity v_m:

$$v_m = \frac{1}{a+b} \tag{9}$$

The specific surface are S_{BET} can be then calculated from the following equation:

$$S_{BET} = \frac{v_m N s}{V} \tag{10}$$

Where N is the Avogadro number (6.02×10^{23}), s is the effective cross-sectional area of N_2 ($1.62 \times 10^{-19}\, m^2$), and V is the molar volume of N_2 at standard temperature and pressure ($0.224\, m^3$/mol).

Supplementary References

1. Wang, F., et al. Progress Report on Phase Separation in Polymer Solutions. *Adv. Mater.* **31**, 1806733 (2019).
2. Gedde, U. W., Hedenqvist, M. S. Polymer Solutions. In: Fundamental Polymer Science. (Springer, 2019).
3. Boparai, H. K., Joseph, M. & O'Carroll, D. M. Kinetics and thermodynamics of cadmium ion removal by adsorption onto nano zerovalent iron particles. *J. Hazard. Mater.* **186**, 458-465 (2011)
4. Ho, Y.-S. Review of second-order models for adsorption systems. *J. Hazard. Mater.* **136**, 681-689 (2006)
